# Supplementary material for: Protein subcellular relocalization and function of duplicated flagellar calcium binding protein genes in honey bee trypanosomatid parasite
Source: PLoS Genet. 2024 Mar 4;20(3):e1011195. doi: 10.1371/journal.pgen.1011195 (PMC10939215; doi:10.1371/journal.pgen.1011195)
Supplement: S1 Data — (DOCX) [file pgen.1011195.s001.docx]

**Alignment of eight FCaBPs**

TcFCaBP **MGACGSKGS----TSDKGLASDKDG** **KNA**KDRKEAWERIRQAIPREKTAEAKQRRIELFKK 56

TbFCaBP **MG-CSGSKNTTNSKDGAASKGGKDG** KTTADRKVAWERIRCAIPRDKDAESKSRRIELFKQ 59

LdFCaBP1 **MG-CNAT---------KAARKPSED** KTAADRKVAWEKICQRLPRQKTPEDKELHIELFKR 50

CfFCaBP1 **MG-CASSAF----SS----KSKKEG** KNASDRKAAWEGIRERLPRKKTDEDKERRIELFKK 51

CfFCaBP2 **MG-CISSKS----TQ----TGKKEG** KTAAERKAAWEGIRQRLPRRKTAEDKARRIELFKK 51

LsFCaBP1 **MG-CASSTS----SS---KGGKKEG** KSAAERRAVWGSVRQSLPRLKTAVDKERRIALFKE 52

LpFCaBP1 **MG-CASSLF----SS----KSKTED** KTAAERKVAWEKIRERLPRRKTPEDKERRIELFKK 51

LpFCaBP2 **MG-CISSKS----TQ----IGKKEC** KTAAERKAAWEGIRQRLPRRKTPEDKQRRIELFKK 51

** * .. .: *.: :*: .* : :** * * :* ***.

TcFCaBP FDKNETGKLCYDEVYSGCLEVLKLDEFTSRVRDITKRAFDKSRTLGSKLE-NKGSEDFVE 115

TbFCaBP FDTNGTGKLGFREVLDGCYSVLKLDEFTTHLPDIVQRAFDKAKDLGNKVK-GVGEEDLVE 118

LdFCaBP FCQSDTEKLTMEEVYQGCVSILQLDEFTTRLRSIVKRAFTKAKSMGNTSGVGQCDDEVVE 110

CfFCaBP1 FDQNNTGKLSFEETYKGCVEILHLDEFTTRLRDIVKRAFNKAKDMGTKDR-DAGSAQFVE 110

CfFCaBP2 FDKNNTGKLSMEETYEGCVSVLHLDEFTTRLRDIVKRAFNKAKDMGTKDR-DAGSAQFVE 110

LsFCaBP FDKNNSGKLSMEEVYKGCVNVLHLDEFTTRLRDIVKRAFNKAKDMGTKER-NEGSSDFVE 111

LpFCaBP1 FDQNNTNRLSVEEVYEGCVDILHLDEFTTRLRDIVKRAFNKAKEMGTKDR-GAGSDKFVE 110

LpFCaBP2 FDKNNTNRLTLEEVYEGCVSILHLDEFTTRLRDIVKRAFNKAKEMGTKDR-GAGSDKFVE 110

* . : :* *. .** .:*:*****::: .*.:*** *:: :*.. . . ..**

TcFCaBP FLEFRLMLCYIYDFFELTVMFDEIDASGNMLVDEEEFKRAVPKLEAWGAKVEDPAALFKE 175

TbFCaBP FLEFRLMLCYIYDIFELTVMFDTMDKDGSLLLELQEFKEALPKLKEWGVDITDATTVFNE 178

LdFCaBP FLEFRLILCYIYDYLELTVMFDEIDTSGNMLVDAREFKAAVPKMGEWGLVIEDPDTIFKE 170

CfFCaBP1 FLEFRLMLCYIYDYFELTVMFDEIDTSGNMLIDAKEFKAAVPKIAEWGLKINDPDAVFKK 170

CfFCaBP2 FLEFRLMLCYIYDYFELTVMFDEIDTSGNMLIDAKEFKKAAPKITEWGVKITDADAVFKE 170

LsFCaBP FLEFRLLLCYIYDYFELTVMFDEIDTSGNMLIDANEFQKAVPKIAEWGLEISDPDAVFKE 171

LpFCaBP1 FLEFRLMLCYIYDYFELTVMFDEIDTSGNMLIDAKEFKKAVPRIQEWGVKIEDPDAVFKE 170

LpFCaBP2 FLEFRLMLCYIYDYFELTVMFDEIDTSGNMLIDAKEFKKAVPRIQEWGVKIEDPDAVFKE 170

******:****** :******* :* .*.:*:: .**: * *:: ** : * ::*::

TcFCaBP LDKNGTGSVTFDEFAAWASAVKLDADGDPDNVPESA---- 211

TbFCaBP IDTNGSGVVTFDEFSCWAVTKKLQVCGDPDGEENGANEGN 218

LdFCaBP IDDNGSGQVPFDELAAWASRSSAGH--------------- 195

CfFCaBP1 IDDNGSGQVTFDEFAAWAAAQKLDADENPGNTE------- 203

CfFCaBP2 IDDNGSGQVTFDEFAAWASARKLDADGDPDNT-------- 202

LsFCaBP IDDNGSGQITFDEFAVWAAAHKLDADGDPDNTAS------ 205

LpFCaBP1 IDNNGSGQITFDEFAAWAAAHKLDADENPDNTE------- 203

LpFCaBP2 IDSNGSGQMTFDEFAAWASAHKLDADGDPDNMAA------ 204

:* **:* : ***:: ** .

**Alignment of 11 FCaBPs**

TcFCaBP MGACGSKGS----TSDKGLASDKDG KNAKDRKEAWERIRQAIPREKTAEAKQRRIELFKK 56

TbFCaBP MG-CSGSKNTTNSKDGAASKGGKDG KTTADRKVAWERIRCAIPRDKDAESKSRRIELFKQ 59

LdFCaBP1 MG-CNATK---------AARKPSED KTAADRKVAWEKICQRLPRQKTPEDKELHIELFKR 50

CfFCaBP1 MG-CASSAF----SS----KSKKEG KNASDRKAAWEGIRERLPRKKTDEDKERRIELFKK 51

CfFCaBP2 MG-CISSKS----TQ----TGKKEG KTAAERKAAWEGIRQRLPRRKTAEDKARRIELFKK 51

LepFCaBP1 MG-CASSAF----SS----KSKKEG KTAAERKAAWESIRQRLPRQKTAEDKERRIELFKK 51

LepFCaBP2-A MG-CTSSKS----TQ----TGKKEG KTAAERKAAWESIRQRLPRQKTAEDKERRIELFKK 51

LepFCaBP2-B MG-CTSSKS----TQ----TGKKEG KTAAERKAAWESIRQRLPRQKTAEDKERRIELFKK 51

LsFCaBP1 MG-CASSTS----SS---KGGKKEG KSAAERRAVWGSVRQSLPRLKTAVDKERRIALFKE 52

LpFCaBP1 MG-CASSLF----SS----KSKTED KTAAERKVAWEKIRERLPRRKTPEDKERRIELFKK 51

LpFCaBP2 MG-CISSKS----TQ----IGKKEC KTAAERKAAWEGIRQRLPRRKTPEDKQRRIELFKK 51

** * .. .: *.: :*: .* : :** * * :* ***.

TcFCaBP FDKNETGKLCYDEVYSGCLEVLKLDEFTSRVRDITKRAFDKSRTLGSKLE-NKGSEDFVE 115

TbFCaBP FDTNGTGKLGFREVLDGCYSVLKLDEFTTHLPDIVQRAFDKAKDLGNKVK-GVGEEDLVE 118

LdFCaBP1 FCQSDTEKLTMEEVYQGCVSILQLDEFTTRLRSIVKRAFTKAKSMGNTSGVGQCDDEVVE 110

CfFCaBP1 FDQNNTGKLSFEETYKGCVEILHLDEFTTRLRDIVKRAFNKAKDMGTKDR-DAGSAQFVE 110

CfFCaBP2 FDKNNTGKLSMEETYEGCVSVLHLDEFTTRLRDIVKRAFNKAKDMGTKDR-DAGSAQFVE 110

LepFCaBP1 FDQNGSGKLTFEETYKGCVSVLHLDEFTTRLRDIVKRAFNKAKDMGTKER-NSGSEQFVE 110

LepFCaBP2-A FDQNGSGKLTFEETYKGCVSVLHLDEFTTRLRDIVKRAFNKAKDMGTKER-NSGSEQFVE 110

LepFCaBP2-B FDQNGSGKLTFEETYKGCVSVLHLDEFTTRLRDIVKRAFNKAKDMGTKER-NSGSEQFVE 110

LsFCaBP1 FDKNNSGKLSMEEVYKGCVNVLHLDEFTTRLRDIVKRAFNKAKDMGTKER-NEGSSDFVE 111

LpFCaBP1 FDQNNTNRLSVEEVYEGCVDILHLDEFTTRLRDIVKRAFNKAKEMGTKDR-GAGSDKFVE 110

LpFCaBP2 FDKNNTNRLTLEEVYEGCVSILHLDEFTTRLRDIVKRAFNKAKEMGTKDR-GAGSDKFVE 110

* . : :* *. .** .:*:*****::: .*.:*** *:: :*.. . . ..**

TcFCaBP FLEFRLMLCYIYDFFELTVMFDEIDASGNMLVDEEEFKRAVPKLEAWGAKVEDPAALFKE 175

TbFCaBP FLEFRLMLCYIYDIFELTVMFDTMDKDGSLLLELQEFKEALPKLKEWGVDITDATTVFNE 178

LdFCaBP1 FLEFRLILCYIYDYLELTVMFDEIDTSGNMLVDAREFKAAVPKMGEWGLVIEDPDTIFKE 170

CfFCaBP1 FLEFRLMLCYIYDYFELTVMFDEIDTSGNMLIDAKEFKAAVPKIAEWGLKINDPDAVFKK 170

CfFCaBP2 FLEFRLMLCYIYDYFELTVMFDEIDTSGNMLIDAKEFKKAAPKITEWGVKITDADAVFKE 170

LepFCaBP1 FLEFRLMLCYIYDYFELTIMFDEIDTSGNMLIDAKEFKKAAPKIAEWGVKISDPDAVFKE 170

LepFCaBP2-A FLEFRLMLCYIYDYFELTIMFDEIDTSGNMLIDAKEFKKAAPKIAEWGVKISDPDAVFKE 170

LepFCaBP2-B FLEFRLMLCYIYDYFELTIMFDEIDTSGNMLIDAKEFKKAAPKIAEWGVKISDPDAVFKE 170

LsFCaBP1 FLEFRLLLCYIYDYFELTVMFDEIDTSGNMLIDANEFQKAVPKIAEWGLEISDPDAVFKE 171

LpFCaBP1 FLEFRLMLCYIYDYFELTVMFDEIDTSGNMLIDAKEFKKAVPRIQEWGVKIEDPDAVFKE 170

LpFCaBP2 FLEFRLMLCYIYDYFELTVMFDEIDTSGNMLIDAKEFKKAVPRIQEWGVKIEDPDAVFKE 170

******:****** :***:*** :* .*.:*:: .**: * *:: ** : * ::*::

TcFCaBP LDKNGTGSVTFDEFAAWASAVKLDADGDPDNVPESA---- 211

TbFCaBP IDTNGSGVVTFDEFSCWAVTKKLQVCGDPDGEENGANEGN 218

LdFCaBP1 IDDNGSGQVPFDELAAWASRSSAGH--------------- 195

CfFCaBP1 IDDNGSGQVTFDEFAAWAAAQKLDADENPGNTE------- 203

CfFCaBP2 IDDNGSGQVTFDEFAAWASARKLDADGDPDNT-------- 202

LepFCaBP1 IDDNGSGQITFDEFAAWASAHKLDVDGDPDNAE------- 203

LepFCaBP2-A IDDNGSGQITFDEFAAWASAHKLDVDGDPDNVAA------ 204

LepFCaBP2-B IDDNGSGQITFDEFAAWASAHKLDVDGDPDNAE------- 203

LsFCaBP1 IDDNGSGQITFDEFAVWAAAHKLDADGDPDNTAS------ 205

LpFCaBP1 IDNNGSGQITFDEFAAWAAAHKLDADENPDNTE------- 203

LpFCaBP2 IDSNGSGQMTFDEFAAWASAHKLDADGDPDNMAA------ 204

:* **:* : ***:: ** .

>TcFCaBP

MGACGSKGSTSDKGLASDKDGKNAKDRKEAWERIRQAIPREKTAEAKQRRIELFKKFDKNETGKLCYDEV

YSGCLEVLKLDEFTSRVRDITKRAFDKSRTLGSKLENKGSEDFVEFLEFRLMLCYIYDFFELTVMFDEID

ASGNMLVDEEEFKRAVPKLEAWGAKVEDPAALFKELDKNGTGSVTFDEFAAWASAVKLDADGDPDNVPES

A

ATGGGTGCTTGTGGGTCGAAGGGCTCGACGAGCGACAAGGGGTTGGCGAGCGATAAGGACGGCAAGAACGCCA

AGGACCGCAAGGAAGCGTGGGAGCGCATTCGCCAGGCGATTCCTCGTGAGAAGACCGCCGAGGCAAAACA

GCGCCGCATCGAGCTCTTCAAGAAGTTCGACAAGAACGAGACCGGGAAGCTGTGCTACGATGAGGTGTAC

AGCGGCTGCCTCGAGGTGCTGAAGTTGGACGAGTTCACGTCGCGAGTGCGCGACATCACGAAGCGTGCAT

TCGACAAGTCGAGGACCCTGGGCAGCAAGCTGGAGAACAAGGGCTCCGAGGACTTTGTTGAATTTCTGGA

GTTCCGTCTGATGCTGTGCTACATCTACGACTTCTTCGAGCTGACGGTGATGTTCGACGAGATTGACGCC

TCCGGCAACATGCTGGTCGACGAGGAGGAGTTCAAGCGCGCCGTGCCCAAGCTTGAGGCGTGGGGCGCCA

AGGTCGAGGATCCCGCGGCGCTGTTCAAGGAGCTCGATAAGAACGGCACTGGGTCCGTGACGTTCGACGA

GTTTGCTGCGTGGGCTTCTGCAGTCAAACTGGACGCCGACGGCGACCCGGACAACGTGCCGGAGAGCGCG

TGA

>TbFCaBP

MGCSGSKNTTNSKDGAASKGGKDGKTTADRKVAWERIRCAIPRDKDAESKSRRIELFKQFDTNGTGKLGF

REVLDGCYSVLKLDEFTTHLPDIVQRAFDKAKDLGNKVKGVGEEDLVEFLEFRLMLCYIYDIFELTVMFD

TMDKDGSLLLELQEFKEALPKLKEWGVDITDATTVFNEIDTNGSGVVTFDEFSCWAVTKKLQVCGDPDGE

ENGANEGN

ATGGGTTGCTCAGGATCGAAGAATACAACGAACTCCAAGGATGGTGCCGCCAGTAAGGGTGGAAAGGACG

GGAAAACTACTGCGGACCGGAAGGTTGCGTGGGAGCGTATTCGGTGTGCGATTCCCCGTGACAAGGATGC

TGAGTCGAAAAGCCGTCGCATTGAACTCTTCAAGCAGTTTGACACGAACGGGACTGGAAAACTCGGCTTC

AGGGAGGTGCTTGATGGTTGCTACAGTGTACTCAAGTTGGATGAGTTCACGACTCACCTTCCTGACATTG

TCCAGCGTGCGTTTGACAAAGCGAAGGACCTCGGCAACAAGGTAAAGGGTGTGGGTGAAGAGGATCTTGT

GGAGTTCCTTGAATTCCGCCTGATGCTTTGCTACATCTATGATATCTTTGAGTTGACTGTAATGTTCGAC

ACCATGGATAAGGATGGAAGTTTGTTGCTTGAGCTACAGGAGTTCAAGGAAGCCTTGCCGAAATTGAAGG

AGTGGGGTGTTGATATCACTGACGCTACCACTGTGTTTAATGAGATCGACACTAATGGATCTGGTGTTGT

GACATTCGATGAATTCTCATGCTGGGCTGTCACTAAAAAGTTGCAGGTATGTGGCGATCCTGATGGTGAG

GAGAACGGAGCAAATGAAGGTAATTGA

>LiFCaBP1

MGCNATKAARKPSEDKTAAERKVAWEKICQRLPRQKTPEDKELRIELFKRFCQSDTEKLTMEEVYQGCVSILQLDEFTTRLRSIVKRAFTKAKSMGNTAGVGQCDDEFVEFLEFRLMLCYIYDYLELTVMFEEIDTSGNMLVDAREFKAAVPKMGEWGLVIEDPDTIFKEIDDNGSGQVPFDELAAWATARKLDADEQANNSE

ATGGGCTGCAACGCTACCAAGGCTGCCCGGAAGCCCAGCGAGGACAAGACTGCCGCTGAGCGCAAGGTCG

CGTGGGAGAAAATTTGTCAGCGCCTGCCCCGCCAGAAGACGCCCGAGGACAAGGAGCTCCGCATCGAGCT

TTTTAAGAGGTTCTGCCAGAGCGACACGGAAAAGCTCACGATGGAGGAGGTCTACCAGGGCTGTGTGAGC

ATCCTCCAGCTCGATGAGTTTACAACGCGCCTGCGCAGCATCGTGAAACGTGCCTTCACTAAGGCAAAGA

GCATGGGCAACACCGCCGGCGTCGGCCAATGCGACGACGAGTTTGTCGAATTTCTTGAGTTTCGGCTGAT

GCTGTGCTACATATACGACTACCTCGAGCTCACCGTCATGTTTGAAGAGATCGACACCTCCGGCAACATG

CTGGTGGACGCGAGGGAGTTCAAGGCCGCCGTGCCGAAGATGGGTGAGTGGGGCCTTGTCATTGAGGATC

CGGACACAATCTTCAAGGAAATCGACGACAACGGCTCCGGCCAGGTGCCCTTTGACGAGCTCGCTGCGTG

GGCCACTGCCCGCAAGCTTGATGCTGACGAGCAGGCTAACAACTCTGAGTAA

>LbFCaBP1

MGCNTSKAAPKSGEDRTAAERKAAWEKIRQSLPRQRTPEDKERHVLLFKKFDNSESGKLTMEEVYQGCVD

ILQLDEFTTRLRDIVQRAFSKAKSMGNTADDGQESSDYVELLEFRLMLCYIYDYFELTVMFDEIDTSGNM

LVSAKEFKAALPRIGEWGVAIEDPDKIFKEIDTNSTGQVTFDEFAAWATGCKLQSDADAMKSE

ATGGGCTGCAACACTTCCAAGGCTGCTCCGAAGTCTGGAGAGGATAGGACTGCCGCCGAGCGCAAGGCTG

CGTGGGAGAAGATCCGCCAGAGCCTACCCCGCCAGAGGACCCCTGAGGACAAGGAGCGCCACGTATTGTT

GTTCAAGAAGTTCGATAATAGCGAATCTGGGAAGCTCACAATGGAGGAGGTCTACCAGGGCTGCGTGGAT

ATTCTTCAGCTTGATGAGTTCACAACGCGCCTGCGCGACATCGTGCAGAGAGCCTTCAGCAAGGCCAAAA

GCATGGGCAACACCGCTGACGACGGCCAGGAAAGCTCTGACTACGTTGAGCTCTTGGAGTTCCGCCTGAT

GCTGTGCTACATCTACGACTACTTCGAGCTCACCGTCATGTTCGACGAGATTGACACCTCCGGCAATATG

CTGGTGAGCGCGAAAGAGTTCAAGGCTGCCCTGCCGAGAATTGGTGAGTGGGGCGTTGCCATCGAGGATC

CAGACAAAATATTCAAGGAGATCGACACGAACAGCACTGGGCAGGTGACATTTGACGAATTCGCTGCGTG

GGCCACTGGATGCAAGCTTCAATCTGACGCAGATGCTATGAAGTCCGAGTAG

>LmFCaBP1

MGCNATKAAGKSGEGKTAAERKVAWEKICQNLPRQRTPEDKERRSDLFKKFSQNDTEKLTMEEVYQGCVRILQLDEFTTRLHDIVKRAFNKAKSMANTAGDCQGDDEFVEFLEFRLMLCYIYDYFKLTVMFDEIDTSGNMLVDAKELKAAVPKIGEWGLVIEDPDTVFKQIDDNGSGQVSFNEFASWATARKLDADEEANDSE

ATGGGCTGTAACGCTACCAAGGCTGCCGGCAAGTCCGGCGAGGGCAAGACCGCCGCTGAGCGCAAGGTTG

CGTGGGAGAAAATTTGTCAGAACCTGCCTCGCCAGAGGACGCCTGAGGACAAGGAACGCCGCAGCGATCT

TTTCAAGAAGTTTTCCCAGAACGACACGGAAAAGCTCACGATGGAGGAAGTCTATCAAGGCTGTGTACGT

ATCCTCCAGCTCGATGAGTTCACGACGCGCCTGCACGACATTGTGAAGCGTGCCTTCAACAAGGCAAAGA

GCATGGCGAACACCGCCGGCGACTGCCAGGGCGACGACGAGTTTGTCGAATTTCTTGAGTTTCGGCTGAT

GCTGTGCTACATATACGACTACTTCAAGCTCACCGTTATGTTTGACGAGATCGACACCTCCGGCAACATG

CTGGTGGACGCGAAGGAGCTCAAGGCCGCCGTGCCGAAGATCGGTGAGTGGGGCCTTGTCATTGAGGATC

CAGACACAGTCTTCAAGCAAATCGACGACAACGGCTCCGGCCAGGTGTCCTTTAACGAGTTCGCTTCGTG

GGCGACTGCCCGCAAGCTTGATGCTGACGAGGAGGCTAACGACTCTGAGTAG

>LdFCaBP1

MGCNATKAARKPSEDKTAADRKVAWEKICQRLPRQKTPEDKELHIELFKRFCQSDTEKLTMEEVYQGCVS

ILQLDEFTTRLRSIVKRAFTKAKSMGNTSGVGQCDDEVVEFLEFRLILCYIYDYLELTVMFDEIDTSGNM

LVDAREFKAAVPKMGEWGLVIEDPDTIFKEIDDNGSGQVPFDELAAWASRSSAGH

ATGGGCTGCAACGCTACCAAGGCTGCCCGGAAGCCCAGCGAGGACAAGACTGCCGCTGAGCGCAAGGTCG

CGTGGGAGAAAATTTGTCAGCGCCTGCCCCGCCAGAAGACGCCCGAGGACAAGGAGCTCCGCATCGAGCT

TTTTAAGAGGTTCTGCCAGAGCGACACGGAAAAGCTCACGATGGAGGAGGTCTACCAGGGCTGTGTGAGC

ATCCTCCAGCTCGAGGAGTTTACAACGCGCCTGCGCAGCATCGTGAAACGTGCCTTCACTAAGGCAAAGA

GCATGGGCAACACCGCCGGCGTCGGCCAATGCGACGACGAGTTTGTCGAATTTCTTGAGTTTCGGCTGAT

GCTGTGCTACATATACGACTACCTCGAGCTCACCGTCATGTTTGACGAGATCGACACCTCCGGCAACATG

CTGGTGGACGCGAGGGAGTTCAAGGCCGCCGTGCCGAAGATGGGTGAGTGGGGCCTTGTCATTGAGGATC

CGGACACAATCTTCAAGGAAATCGACGACAACGGCTCCGGCCAGGTGTCCTTTGACGAGCTCGCTGCGTG

GGCCACTGCCCGCAAGCTTGATGCTGACGAGCAGGCTAACAACTCTGAGTAA

>CfFCaBP1

MGCASSAFSSKSKKEGKNASDRKAAWEGIRERLPRKKTDEDKERRIELFKKFDQNNTGKLSFEETYKGCVEILHLDEFTTRLRDIVKRAFNKAKDMGTKDRDAGSAQFVEFLEFRLMLCYIYDYFELTVMFDEIDTSGNMLIDAKEFKAAVPKIAEWGLKINDPDAVFKKIDDNGSGQVTFDEFAAWAAAQKLDADENPGNTE

ATGGGTTGCGCTTCTTCCGCTTTTTCCTCCAAGTCCAAGAAGGAGGGCAAGAACGCCAGCGATCGCAAGGCCGCGTGGGAGGGCATTCGCGAGCGTCTGCCGCGCAAGAAGACTGACGAGGACAAGGAGCGCCGCATTGAGCTCTTCAAGAAGTTCGACCAGAACAACACCGGCAAGCTGTCGTTTGAGGAGACGTACAAGGGATGCGTAGAGATTCTGCACCTGGACGAGTTCACCACTCGTCTGCGCGACATCGTCAAGCGCGCCTTCAACAAGGCCAAGGACATGGGCACGAAGGACCGCGATGCGGGCAGTGCTCAGTTCGTGGAGTTCCTCGAGTTCCGTCTGATGCTCTGCTACATCTACGACTACTTTGAGCTGACCGTGATGTTTGACGAGATCGACACTTCTGGCAACATGCTGATCGACGCGAAGGAGTTCAAGGCCGCCGTCCCTAAGATTGCGGAGTGGGGATTGAAGATTAATGACCCTGATGCCGTCTTCAAGAAGATCGACGACAACGGCTCCGGTCAGGTGACCTTCGACGAGTTTGCAGCGTGGGCGGCGGCCCAGAAACTCGACGCTGACGAGAACCCTGGCAACACGGAATAA

>CfFCaBP2

MGCISSKSTQTGKKEGKTAAERKAAWEGIRQRLPRRKTAEDKARRIELFKKFDKNNTGKLSMEETYEGCVSVLHLDEFTTRLRDIVKRAFNKAKDMGTKDRDAGSAQFVEFLEFRLMLCYIYDYFELTVMFDEIDTSGNMLIDAKEFKKAAPKITEWGVKITDADAVFKEIDDNGSGQVTFDEFAAWASARKLDADGDPDNTAA

ATGGGGTGCATTTCTTCCAAGTCTACTCAGACCGGCAAGAAGGAGGGCAAGACTGCCGCCGAGCGCAAGGCCGCGTGGGAGGGCATTCGCCAGCGCCTGCCGCGCCGCAAGACTGCCGAGGACAAGGCACGCCGCATTGAGCTCTTCAAGAAGTTCGATAAGAACAACACCGGCAAGCTCAGCATGGAGGAGACGTACGAGGGCTGCGTGTCTGTGTTGCACCTGGACGAGTTCACCACTCGTCTGCGCGACATCGTCAAGCGCGCCTTCAACAAGGCCAAGGACATGGGCACGAAGGACCGCGATGCGGGCAGCGCTCAGTTCGTGGAGTTCCTCGAGTTCCGTCTGATGCTCTGCTACATCTACGACTACTTCGAGCTGACCGTGATGTTTGACGAGATCGACACTTCTGGCAACATGCTGATCGACGCGAAGGAGTTCAAGAAGGCTGCTCCCAAGATTACGGAGTGGGGCGTGAAGATCACCGATGCCGATGCCGTCTTCAAGGAGATCGACGACAACGGCTCCGGTCAGGTGACCTTCGACGAGTTTGCGGCGTGGGCGAGCGCGCGCAAGCTCGACGCCGACGGCGATCCGGATAACACCGCTGCTTAA

>LsFCaBP1

MGCASSTSSSKGGKKEGKSAAERRAVWGSVRQSLPRLKTAVDKERRIALFKEFDKNNSGKLSMEEVYKGC

VNVLHLDEFTTRLRDIVKRAFNKAKDMGTKERNEGSSDFVEFLEFRLLLCYIYDYFELTVMFDEIDTSGN

MLIDANEFQKAVPKIAEWGLEISDPDAVFKEIDDNGSGQITFDEFAVWAAAHKLDADGDPDNTAS

ATGGGTTGCGCCTCTTCCACATCCTCTTCAAAGGGCGGCAAGAAGGAGGGCAAGAGCGCCGCAGAGCGCA

GAGCTGTGTGGGGCAGCGTTCGCCAGAGTCTGCCGCGCCTGAAGACTGCAGTGGATAAGGAGCGCCGTAT

TGCACTCTTTAAGGAGTTCGATAAGAACAACAGTGGTAAGCTCAGCATGGAGGAGGTGTACAAGGGTTGC

GTGAATGTGCTGCATCTGGATGAGTTCACGACTCGCCTGCGCGACATCGTGAAGCGCGCCTTCAACAAGG

CCAAGGATATGGGAACGAAGGAGCGCAACGAGGGCAGCTCGGACTTCGTGGAGTTCCTGGAGTTCCGTCT

GTTGCTGTGCTACATCTACGACTACTTTGAGCTGACGGTGATGTTCGATGAGATCGACACTTCTGGCAAC

ATGCTGATCGATGCGAATGAGTTCCAGAAGGCTGTCCCGAAGATCGCGGAGTGGGGTTTGGAGATTTCCG

ACCCCGATGCCGTGTTCAAGGAGATCGACGACAACGGCTCTGGTCAGATCACGTTTGACGAGTTCGCCGT

ATGGGCTGCCGCCCACAAGCTGGATGCTGATGGCGATCCGGATAATACCGCTTCGTAA

>CeFaBP1

MGCASSVFSSKSKKEGKSAAERKVAWGRIRERLPRKKTPEDKERRIELFKKFDQNGTGKLSFEETYRGCVDILHLDEFTTRLRDIVKRAFSKAKDMGTKDRGAGSDQFVEFLEFRLMLCYIYDYFELTVMFDEIDTSGNMLIDAKEFKAAVPKIAEWGLKIEDPDAVFKQIDDNGSGQITFDEFAAWAAARKLDADENPNNAE

ATGGGCTGCGCCTCCTCCGTATTTTCCTCCAAGTCAAAGAAAGAGGGCAAGAGCGCCGCCGAACGTAAAGTGGCGTGGGGGAGGATCCGCGAGCGTCTGCCGCGCAAGAAGACTCCTGAGGACAAAGAGCGCCGCATTGAACTCTTCAAGAAGTTCGATCAGAACGGCACCGGTAAACTTTCGTTTGAGGAGACGTACAGGGGGTGCGTAGACATTCTGCATCTGGATGAGTTCACGACTCGCCTGCGCGACATCGTGAAGCGCGCCTTCAGCAAGGCCAAAGACATGGGCACGAAGGACCGCGGCGCGGGTAGCGACCAGTTTGTGGAGTTCCTTGAGTTCCGCCTGATGCTGTGCTACATCTACGACTACTTTGAGCTGACTGTGATGTTCGACGAGATCGATACTTCTGGTAACATGCTTATCGACGCGAAGGAGTTCAAGGCCGCGGTCCCCAAGATTGCGGAGTGGGGCCTCAAGATTGAAGATCCCGACGCCGTGTTCAAGCAGATCGATGACAACGGCTCTGGCCAGATCACGTTCGACGAGTTCGCTGCGTGGGCAGCTGCGCGCAAGCTCGATGCTGACGAGAACCCCAACAACGCTGAGTGA

>CeFCaBP2

MGCISSKSTSSKKEGKNAAERKVAWEGIRQRLPRRKTPEDKARRIELFKQFDKNNTGKLSLEEVYNGCVTVLHLDEFTTRLRDIVKRAFSKAKDMGTKDRGAGSDQFVEFLEFRLMLCYIYDYFELTVMFDEIDTSGNMLIDAKEFKKAAQKITEWGVKIEDPDAVFKQIDDNGSGQITFDEFAAWASAHKLDADGDPDNV

ATGGGGTGCATTTCGTCTAAGTCGACTTCGTCGAAGAAGGAAGGCAAGAACGCCGCGGAGCGCAAGGTCGCATGGGAGGGCATTCGCCAGCGTCTACCTCGCCGCAAGACTCCTGAGGACAAGGCACGCCGCATTGAGCTCTTCAAACAGTTCGACAAAAACAACACCGGTAAGCTCAGCCTGGAGGAGGTGTACAATGGCTGTGTCACTGTGCTGCATCTGGATGAGTTCACGACTCGCCTGCGCGACATCGTGAAGCGCGCCTTCAGCAAGGCCAAAGACATGGGCACGAAGGACCGCGGCGCGGGTAGCGACCAGTTTGTGGAGTTCCTTGAGTTCCGCCTGATGCTGTGCTACATCTACGACTACTTTGAGCTGACTGTGATGTTCGACGAGATCGATACTTCTGGCAACATGCTGATCGACGCGAAGGAATTTAAGAAGGCTGCTCAGAAGATCACGGAATGGGGTGTGAAGATTGAAGATCCCGACGCCGTGTTCAAGCAGATCGATGACAACGGCTCTGGCCAGATCACGTTCGACGAGTTTGCTGCGTGGGCGAGCGCGCACAAGCTCGACGCTGATGGCGATCCGGACAATGTGGCTGCGTAA

>CbFCaBP2

MGCISSKSTQSKKEGKTAAERKAAWEGIRHRLPRRKTPEDKARRIELFKKFDKNGTGKLSLEETYEGCVSVLHLDEFTTRLRDIVKRAFNKAKEMGTKDRNAGSDQFVEFLEFRLMLCYIYDYFELTVMFDEIDTSGNMLIDAKEFKKAAPKITEWGVKITDPDAVFREIDDNGSGQITFDEFAAWASAHKLDADGDPDNV

ATGGGGTGCATCTCTTCTAAGTCCACTCAGTCGAAGAAGGAGGGCAAGACTGCTGCGGAGCGCAAAGCAGCGTGGGAGGGCATCCGCCACCGCCTGCCTCGCCGCAAGACTCCCGAAGATAAGGCACGCCGTATCGAGCTCTTTAAGAAGTTTGACAAGAACGGTACCGGCAAGCTCAGCTTGGAGGAGACGTACGAGGGCTGCGTATCTGTACTGCACCTGGATGAGTTCACGACTCGTCTGCGCGACATCGTGAAGCGCGCCTTCAACAAGGCAAAGGAAATGGGAACGAAGGACCGCAATGCTGGCAGCGACCAGTTCGTGGAGTTTCTGGAGTTCCGCCTGATGCTGTGCTACATCTACGACTACTTTGAGCTGACAGTGATGTTCGACGAGATTGATACTTCTGGCAACATGCTGATCGACGCGAAGGAGTTCAAGAAGGCTGCTCCGAAGATCACGGAATGGGGTGTGAAGATTACCGACCCCGATGCCGTGTTCAGGGAGATCGACGACAACGGCTCTGGCCAGATCACGTTCGACGAGTTTGCTGCTTGGGCGAGCGCGCACAAGCTGGACGCCGATGGTGATCCGGACAACGTGGCTGCGTAA

>CbFCaBP1

MGCASSAFSSKSKEGKTAAERKVAWGRIRERLPRKKTPEDKERRIELFKKFDVNGTGKLSFEETYRGCIDILHLDEFTTRVRDIVKRAFNKARDMGTKDRNAGSDQFVEFLEFRLMLCYIYDYFELTVMFDEIDTSGNMLIDAKEFKAAVPKIAEWGLKITDPDAVFRKIDGNGSGQITFDEFAAWAAAHRLDADENVYNTK

ATGGGCTGCGCCAGTTCCGCTTTCTCATCCAAGTCCAAGGAGGGCAAGACTGCTGCGGAGCGCAAGGTCGCGTGGGGGAGGATTCGTGAGCGCCTGCCGCGGAAGAAGACTCCCGAGGACAAGGAGCGTCGCATCGAGCTTTTCAAGAAGTTCGATGTGAACGGGACTGGCAAGCTGTCGTTCGAGGAGACGTACAGGGGCTGCATAGATATTCTGCACCTGGATGAGTTCACGACTCGTGTGCGCGACATCGTGAAGCGCGCCTTCAACAAGGCAAGGGACATGGGAACGAAGGACCGCAATGCTGGCAGCGACCAGTTCGTGGAGTTTCTGGAGTTCCGCCTGATGCTGTGCTACATCTACGACTACTTTGAGCTGACAGTGATGTTCGACGAGATTGATACTTCTGGCAACATGCTGATCGACGCGAAGGAATTCAAGGCTGCCGTCCCGAAGATTGCGGAATGGGGTCTGAAGATCACCGACCCCGATGCCGTGTTCAGGAAGATTGACGGCAACGGCTCTGGCCAGATCACGTTCGACGAGTTTGCTGCGTGGGCTGCTGCGCACCGTCTCGATGCCGATGAGAACGTCTACAACACCAAGTAA

>LpFCaBP1

MGCASSLFSSKSKTEDKTAAERKVAWEKIRERLPRRKTPEDKERRIELFKKFDQNNTNRLSVEEVYEGCVDILHLDEFTTRLRDIVKRAFNKAKEMGTKDRGAGSDKFVEFLEFRLMLCYIYDYFELTVMFDEIDTSGNMLIDAKEFKKAVPRIQEWGVKIEDPDAVFKEIDNNGSGQITFDEFAAWAAAHKLDADENPDNTE

ATGGGCTGTGCTTCTTCCCTGTTTTCCTCCAAGTCCAAGACAGAGGATAAAACGGCTGCGGAACGCAAGGTCGCGTGGGAGGGGATCCGCGAGCGTCTGCCTCGCCGCAAGACCCCTGAGGACAAGGAGCGCCGCATTGAGCTCTTTAAGAAGTTCGATCAGAACAACACCAACAGGCTCACCCTTGAGGAGGTCTACGAGGGCTGCGTAGATATTCTGCACCTGGACGAGTTTACGACTCGTCTGCGTGACATTGTGAAGCGCGCCTTCAACAAGGCCAAGGAGATGGGAACGAAGGATCGTGGCGCAGGCAGCGACAAGTTTGTGGAGTTCCTTGAGTTCCGCCTGATGCTGTGCTACATCTACGACTACTTCGAGCTGACTGTGATGTTCGACGAGATCGACACTTCTGGCAATATGCTGATCGACGCAAAGGAGTTCAAGAAGGCTGTTCCAAGGATCCAGGAATGGGGTGTGAAGATTGAGGACCCCGATGCCGTGTTTAAGGAGATCGACAGCAACGGCTCTGGCCAGATCACGTTTGACGAGTTTGCCGCATGGGCGGCAGCGCACAAGCTTGATGCCGATGAGAACCCTGACAACACAGAATAA

>LpFCaBP2

MGCISSKSTQIGKKECKTAAERKAAWEGIRQRLPRRKTPEDKQRRIELFKKFDKNNTNRLTLEEVYEGCVSILHLDEFTTRLRDIVKRAFNKAKEMGTKDRGAGSDKFVEFLEFRLMLCYIYDYFELTVMFDEIDTSGNMLIDAKEFKKAVPRIQEWGVKIEDPDAVFKEIDSNGSGQMTFDEFAAWASAHKLDADGDPDNMAA

ATGTTCCTCTTTTTTTTTTTTCTGTTGGAAGAGCTGTGTCGTCTAGTTTGGTCGTCCGCCCCCCCGTCTAGTTCCCGCTTATTCCCCTCCCCTGTTTGCCCGCCGTGTGTGGCCTCTCTCTACACTCTCGCCTCCCTCCGCTGTAGTTTTTTTCGCGCGCGCACACAATTCTCTCTCCCCAGAGAGAGCCACTATTCTCACTGTGCAAAACTGGGCTGCATCTCTTCAAAGTCTACTCAGATTGGCGAGAGGGAGTGCAAGACGGCTGCGGAGCGCAAGGCCGCGTGGGAGGGGATTCGCGAACGTCTGCCTCGCCGCAAGACCCCTGAGGACAAGGAGCGCCGCATTGAGCTCTTTAAGAAGTTTGATCAGAACAACACCAACAGGCTCACCCTTGAGGAGGTCTACGAGGGCTGCGTGTCTATTCTGCACCTGGACGAGTTTACGACTCGTCTGCGTGACATTGTGAAGCGCGCCTTCAACAAGGCCAAGGAGATGGGAACGAAGGATCGTGGCGCAGGCAGCGACAAGTTTGTGGAGTTCCTTGAGTTCCGCCTGATGCTGTGCTACATCTACGACTACTTCGAGCTGACTGTGATGTTCGACGAGATCGACACTTCTGGCAATATGCTGATCGACGCAAAGGAGTTCAAGAAGGCTGTTCCAAGGATCCAGGAATGGGGTGTGAAGATTGAGGACCCCGATGCCGTGTTTAAGGAGATCGACAGCAATGGCTCTGGCCAGATGACGTTTGACGAGTTTGCCGCATGGGCGGCAGCGCACAAGCTTGACGCCGATGGCGACCCGGACAACATGGCTGCGTAA

>LepFCaBP2-A

MGCTSSKSTQTGKKEGKTAAERKAAWESIRQRLPRQKTAEDKERRIELFKKFDQNGSGKLTFEETYKGCVSVLHLDEFTTRLRDIVKRAFNKAKDMGTKERNSGSEQFVEFLEFRLMLCYIYDYFELTIMFDEIDTSGNMLIDAKEFKKAAPKIAEWGVKISDPDAVFKEIDDNGSGQITFDEFAAWASAHKLDVDGDPDNVAA

ATGGGGTGCACCTCTTCGAAATCTACTCAGACTGGCAAGAAGGAGGGCAAGACTGCCGCGGAGCGTAAGG

CTGCGTGGGAAAGCATCCGCCAGCGTCTGCCCCGCCAGAAGACCGCCGAAGATAAGGAGCGCCGCATTGA

ACTTTTCAAGAAGTTCGATCAGAACGGTTCAGGCAAGTTGACATTCGAAGAGACGTACAAGGGGTGCGTA

TCTGTTCTGCATCTGGATGAGTTCACGACTCGTCTGCGTGATATCGTGAAGCGCGCCTTCAACAAGGCCA

AGGACATGGGAACGAAGGAGCGCAACTCAGGCAGCGAGCAGTTCGTGGAGTTCCTCGAGTTCCGTCTGAT

GCTGTGCTACATCTACGACTACTTTGAGCTAACCATTATGTTCGATGAGATCGACACTTCTGGTAACATG

CTGATCGACGCGAAGGAGTTCAAGAAGGCTGCTCCGAAGATCGCAGAGTGGGGTGTGAAGATCAGCGACC

CTGATGCCGTGTTCAAGGAGATCGACGACAATGGCTCGGGTCAGATCACGTTTGACGAATTCGCCGCCTG

GGCGAGTGCCCACAAGCTCGACGTCGATGGCGATCCGGACAACGTGGCTGCGTAG

>LepFCaBP2-B

MGCTSSKSTQTGKKEGKTAAERKAAWESIRQRLPRQKTAEDKERRIELFKKFDQNGSGKLTFEETYKGCVSVLHLDEFTTRLRDIVKRAFNKAKDMGTKERNSGSEQFVEFLEFRLMLCYIYDYFELTIMFDEIDTSGNMLIDAKEFKKAAPKIAEWGVKISDPDAVFKEIDDNGSGQITFDEFAAWASAHKLDVDGDPDNAE

ATGGGGTGCACCTCTTCGAAATCTACTCAGACTGGCAAGAAGGAGGGCAAGACTGCCGCGGAGCGTAAGG

CTGCGTGGGAAAGCATCCGCCAGCGTCTGCCCCGCCAGAAGACCGCCGAAGATAAGGAGCGCCGCATTGA

ACTTTTCAAGAAGTTCGATCAGAACGGTTCAGGCAAGTTGACATTCGAAGAGACGTACAAGGGGTGCGTA

TCTGTTCTGCATCTGGATGAGTTCACGACTCGTCTGCGTGATATCGTGAAGCGCGCCTTCAACAAGGCCA

AGGACATGGGAACGAAGGAGCGCAACTCAGGCAGCGAGCAGTTCGTGGAGTTCCTCGAGTTCCGTCTGAT

GCTGTGCTACATCTACGACTACTTTGAGCTAACCATTATGTTCGATGAGATCGACACTTCTGGTAACATG

CTGATCGACGCGAAGGAGTTCAAGAAGGCTGCTCCGAAGATCGCAGAGTGGGGTGTGAAGATCAGCGACC

CTGATGCCGTGTTCAAGGAGATCGACGACAATGGCTCGGGTCAGATCACGTTTGACGAATTCGCCGCCTG

GGCGAGTGCCCACAAGCTCGACGTCGATGGCGATCCGGACAACGCCGAATAA

>LepFCaBP1

MGCASSAFSSKSKKEGKTAAERKAAWESIRQRLPRQKTAEDKERRIELFKKFDQNGSGKLTFEETYKGCVSVLHLDEFTTRLRDIVKRAFNKAKDMGTKERNSGSEQFVEFLEFRLMLCYIYDYFELTIMFDEIDTSGNMLIDAKEFKKAAPKIAEWGVKISDPDAVFKEIDDNGSGQITFDEFAAWASAHKLDVDGDPDNAE

ATGGGCTGCGCTTCTTCCGCATTCTCCTCCAAATCCAAGAAGGAGGGCAAGACTGCCGCGGAGCGTAAGG

CTGCGTGGGAAAGCATCCGCCAGCGTCTGCCCCGCCAGAAGACCGCCGAAGATAAGGAGCGCCGCATTGA

ACTTTTCAAGAAGTTCGATCAGAACGGTTCAGGCAAGTTGACATTCGAAGAGACGTACAAGGGGTGCGTA

TCTGTTCTGCATCTGGATGAGTTCACGACTCGTCTGCGTGATATCGTGAAGCGCGCCTTCAACAAGGCCA

AGGACATGGGAACGAAGGAGCGCAACTCAGGCAGCGAGCAGTTCGTGGAGTTCCTCGAGTTCCGTCTGAT

GCTGTGCTACATCTACGACTACTTTGAGCTAACCATTATGTTCGATGAGATCGACACTTCTGGTAACATG

CTGATCGACGCGAAGGAGTTCAAGAAGGCTGCTCCGAAGATCGCAGAGTGGGGTGTGAAGATCAGCGACC

CTGATGCCGTGTTCAAGGAGATCGACGACAATGGCTCGGGTCAGATCACGTTTGACGAATTCGCCGCCTG

GGCGAGTGCCCACAAGCTCGACGTCGATGGCGATCCGGACAACGCCGAATAA
