# Supplementary material for: Protein subcellular relocalization and function of duplicated flagellar calcium binding protein genes in honey bee trypanosomatid parasite
Source: PLoS Genet. 2024 Mar 4;20(3):e1011195. doi: 10.1371/journal.pgen.1011195 (PMC10939215; doi:10.1371/journal.pgen.1011195)
Supplement: S2 Data — (DOCX) [file pgen.1011195.s002.docx]

***Trypanosoma theileri* FCaBPs**

MGLCGSKNSTSSKEGKSAQDRKVAWERIRQAIPREKTPEAKQRRIDLFKKFDKNNSGKLSYNEVYEGCLNVLKLDEFTSRLRDITKRAFNKAKDMGNKVENKGSEDYVEFLEFRLFLCYVYDYFELTVMFDEIDTSGNMLLDENEFKKAAPKLEEWGAKIDDPSKVFKELDKNGSGAVTFDEFAAWASARKLDVDGDPDNVPGNS

ATGGGTTTATGTGGATCTAAGAACAGCACCTCCAGCAAGGAGGGTAAGTCTGCCCAGGACCGCAAGGTTG

CATGGGAGCGCATCCGGCAGGCAATTCCCCGTGAAAAGACACCTGAGGCGAAGCAGCGACGCATCGACTT

GTTTAAGAAGTTCGACAAAAACAATTCTGGGAAGCTGTCTTATAATGAGGTGTATGAAGGCTGTTTGAAT

GTGCTGAAGCTGGATGAATTCACTTCACGGCTACGGGACATCACGAAGCGTGCGTTCAACAAAGCAAAGG

ACATGGGTAACAAGGTGGAGAACAAGGGTTCTGAGGACTATGTTGAATTCCTGGAGTTCCGTTTGTTTCT

GTGCTACGTGTACGACTACTTTGAGCTGACAGTGATGTTCGATGAAATCGACACCTCCGGCAACATGCTG

CTCGATGAGAATGAGTTCAAGAAGGCCGCGCCGAAGCTTGAGGAGTGGGGTGCTAAGATCGATGATCCCA

GCAAAGTGTTCAAGGAGCTGGACAAAAACGGTTCTGGTGCAGTGACGTTCGACGAATTTGCTGCTTGGGC

CTCTGCGCGCAAGCTGGACGTGGACGGTGATCCGGACAACGTTCCAGGCAATTCTTAG

MGSSCSKKEQIKNGFTKKEGKSAQDRKVAWERIRQAIPREKTPEAKQRRADLFKKFDKNNSGKLSYDEVYDGCLNVLKLDEFTTRLRDITKRAFNKAKDMGNKVENKGSEDFVEFLEFRLFLCYVYDYFELTVMFDEIDTSGNMLIDEKEFKKAVPKLEKWGAKIDDPSKVFKELDKNGSGSVTFDEFAAWASARKLDVDGDPDNVPEAPQVPDVPES

ATGGGGTCATCTTGCTCTAAAAAGGAACAAATAAAGAACGGGTTCACCAAGAAGGAGGGTAAGTCTGCCC

AGGACCGCAAGGTTGCATGGGAGCGCATCCGGCAGGCAATTCCCCGTGAAAAGACACCTGAAGCGAAGCA

GCGACGCGCCGACCTGTTTAAGAAGTTCGACAAAAACAATTCTGGGAAGCTGTCTTATGATGAGGTGTAT

GATGGCTGTTTGAATGTGCTGAAGCTGGATGAATTCACTACACGGCTACGGGACATCACGAAGCGTGCGT

TCAACAAGGCAAAGGACATGGGTAACAAGGTGGAGAACAAGGGTTCTGAGGACTTTGTTGAATTCCTGGA

GTTCCGTTTGTTTCTGTGCTACGTGTACGACTACTTTGAGCTGACAGTGATGTTCGATGAAATCGACACC

TCCGGCAACATGCTGATTGATGAGAAGGAGTTCAAGAAGGCCGTGCCGAAGCTTGAAAAGTGGGGTGCTA

AGATCGATGATCCCAGCAAAGTGTTCAAGGAGCTGGACAAAAACGGTTCTGGCTCAGTGACGTTTGACGA

ATTTGCTGCTTGGGCCTCTGCGCGCAAGCTGGACGTGGACGGTGATCCGGACAACGTTCCAGAGGCACCA

CAGGTTCCAGACGTGCCAGAAAGTTCTTAG

MGFCGSKSSTSSKEGKSSQDRKVAWERIRQVIPREKTPEAKQRRIDLFKKFDKNNSGKLSYDEVYEGCLNVLKLDEFTTRLRDITKRAFNRAKDMGNKVANKGSEDFVEFMEFRLFLCYVYDYFDLMVMFDKIDTSGNMLIDEKEFKKAVPKLEKWGAKIDDPSKVFKELDKNGSGSVTFDEFAAWASARKLDVDGDPDNVPGSS

ATGGGGTTTTGTGGATCAAAGAGCAGTACCTCCAGCAAGGAGGGTAAGTCTTCCCAGGACCGCAAGGTTG

CATGGGAGCGTATCCGGCAGGTAATTCCCCGTGAAAAGACACCTGAGGCGAAGCAGCGACGCATCGACTT

GTTTAAGAAGTTCGACAAAAACAATTCTGGGAAGCTGTCTTATGATGAGGTGTATGAAGGTTGTTTGAAT

GTGCTGAAGCTGGATGAATTCACTACACGGCTACGGGACATCACGAAGCGTGCGTTCAACAGGGCAAAGG

ACATGGGTAACAAGGTGGCGAACAAGGGTTCTGAGGACTTTGTTGAATTCATGGAGTTCCGTTTGTTTCT

GTGCTACGTGTACGACTACTTTGATTTGATGGTGATGTTCGATAAAATCGACACCTCCGGCAACATGCTG

ATTGATGAGAAGGAGTTCAAGAAGGCCGTGCCGAAGCTTGAAAAGTGGGGTGCTAAGATCGATGATCCCA

GC

**LpPFRP5**

MTTAASLFERNKRIYLDEESKLHDIVCDDQFLRTFQNWLDQSISKLDFQYERCEQQLSELQQHVSVPKGSFWNSKAIIEHCNLHMAERRLAKRGKELGGDDDGTGDQHVLLPDLIQLVSALQQTKSHSFITAKQRRFIEECEDELKSVVFEPRDLTFITNALQTKLIDDGNTRGVFGDLTAFQNTIEELSPDIDQCEQLLEASIANGEMGLAEDISKRQLDVYEHILRLITDQYPIISNYYSESRNSDRRRRWAVFRMADRDITAVIESKHRQIEACEEDMLKIQEQTTNYNNDDAQQRKRYEADKAESDQFLQQNKEKQQSVWNRVFALFQELQGCSSELATLAEQRRREVERRLQMEEREAGRRSGHESFLQAAAEHAQKLQDTIDNAAAARDVATALNDFVLDGCDSIAAKYDKQQNALGEMLRLVQQHHFKRFSDYYIAASRYLYRKERRLEQIDEEMRSNDMQRELFSDTLNPQAKEYAEANQRLSLQRHEVSQEVMHVRHKLERAERAVTPTLRSLDFANIAYVHPREIVEKMNLSRWSTMLDYRTMLNKSGEDEAELQREAAAIEEMRAELDAQKATSHSQHLLRGATGVRIVTTVPLGATGNKASSKPDAAATAPSSLTCASSVKKQQPSRFVERVYAMLQRGEEPRAGGAAGGGSSSTAAAPKPSSTAAAAGGALAVLPTASLLSPSSLSTRANNNNNDGPLTAGGSMSPAPPPSHIEGATFQALFNYRARAPDELTFEAGQQIICISRAPEEGWFKGVCNQRTGLFPINYVEPVREAASTT

ATGACAACCGCGGCATCGCTGTTTGAGCGCAATAAGCGCATATACCTCGACGAGGAGTCGAAGCTGCACGATATCGTGTGTGACGATCAGTTTCTGCGCACCTTCCAGAATTGGCTGGACCAGAGTATCTCGAAGCTGGATTTCCAGTACGAGCGATGCGAGCAGCAGTTGTCGGAGTTGCAGCAGCATGTGTCGGTCCCGAAGGGTTCCTTCTGGAACAGCAAGGCGATCATTGAGCACTGCAACCTGCATATGGCAGAGCGACGTCTCGCGAAGCGAGGAAAGGAGCTTGGCGGTGACGACGACGGCACGGGCGACCAGCACGTGCTGTTACCGGACCTGATTCAACTTGTGTCGGCGCTGCAGCAGACCAAGTCGCACTCTTTCATCACGGCGAAGCAGCGCCGCTTCATCGAGGAATGCGAGGATGAGCTGAAGTCGGTCGTGTTCGAGCCACGTGATTTGACGTTCATCACAAATGCACTGCAGACAAAGCTGATTGACGACGGCAATACCCGCGGCGTCTTTGGCGACCTCACCGCGTTCCAGAACACGATTGAGGAGCTGTCGCCTGATATCGACCAGTGCGAGCAGCTGTTGGAGGCGAGTATCGCGAATGGCGAGATGGGCCTCGCCGAGGACATCTCGAAGCGGCAGCTGGACGTCTACGAGCACATTCTGCGCCTCATTACAGATCAGTACCCGATCATTTCTAATTACTACTCCGAATCGCGCAACAGCGACCGCCGGCGACGCTGGGCAGTCTTCCGCATGGCGGATCGCGACATCACAGCCGTGATCGAGTCGAAGCATCGCCAAATCGAGGCATGTGAGGAGGACATGCTGAAGATCCAGGAGCAGACGACAAACTACAACAACGACGACGCGCAGCAGCGCAAGCGCTATGAGGCGGATAAGGCCGAATCGGACCAGTTTCTTCAGCAGAACAAGGAGAAGCAGCAGAGCGTGTGGAATCGCGTCTTTGCGCTCTTCCAGGAGCTGCAGGGCTGCTCGAGTGAGCTGGCGACGCTGGCGGAGCAGCGACGGAGGGAAGTGGAGAGACGGCTGCAGATGGAGGAGCGCGAAGCGGGGCGGCGCAGCGGCCACGAGAGCTTCCTGCAAGCCGCGGCCGAGCACGCACAGAAGCTGCAGGACACCATCGACAACGCGGCTGCCGCGCGCGACGTCGCGACGGCGCTCAATGACTTTGTGCTCGACGGCTGCGACAGCATTGCGGCGAAGTACGACAAGCAGCAGAACGCGTTGGGGGAGATGCTGCGGCTAGTGCAGCAGCACCACTTCAAGCGCTTCTCCGACTACTACATCGCAGCCAGTCGCTACCTCTACCGCAAAGAGCGGCGTCTGGAGCAGATTGACGAGGAGATGCGGTCGAACGACATGCAGCGCGAGCTTTTCTCCGACACGCTGAACCCGCAGGCAAAGGAATACGCGGAGGCGAACCAACGACTGTCGCTGCAACGGCACGAGGTGTCGCAGGAGGTGATGCACGTGCGCCACAAGTTGGAGCGGGCAGAACGGGCCGTCACGCCGACGCTGCGCTCGCTTGACTTCGCCAACATCGCCTACGTCCACCCACGCGAGATCGTGGAGAAGATGAACCTGAGTCGCTGGAGCACCATGCTGGACTACCGCACGATGCTGAACAAGTCTGGCGAGGATGAGGCGGAGTTGCAGCGCGAGGCCGCGGCGATCGAGGAGATGCGGGCGGAGCTTGACGCGCAGAAGGCGACGTCGCACAGCCAGCATCTGCTGCGAGGCGCGACTGGGGTGCGGATTGTCACCACCGTACCGCTTGGCGCCACCGGAAACAAGGCAAGCTCGAAGCCGGATGCTGCGGCGACGGCGCCAAGCTCGCTGACGTGTGCGTCCTCCGTGAAAAAGCAGCAACCATCGCGGTTCGTGGAGCGGGTGTATGCAATGCTGCAACGGGGAGAGGAGCCGCGGGCCGGTGGTGCCGCGGGAGGCGGCTCCTCGTCCACCGCCGCCGCCCCGAAGCCGTCCTCCACGGCCGCGGCAGCTGGCGGCGCGTTGGCGGTGCTTCCCACAGCCTCGTTGCTGTCCCCTTCATCCCTCTCCACCCGGGCGAATAACAACAACAACGACGGCCCGCTGACGGCTGGTGGGTCAATGAGCCCAGCCCCGCCACCGTCGCACATCGAAGGCGCCACCTTCCAGGCCCTCTTTAACTACCGCGCACGCGCGCCAGATGAGCTAACCTTTGAAGCAGGGCAGCAGATCATCTGCATTAGTCGCGCACCGGAGGAGGGGTGGTTTAAGGGTGTGTGCAACCAGCGCACAGGGCTGTTTCCCATCAACTACGTGGAGCCGGTGCGCGAGGCGGCAAGCACGACTTGA

**LpBBS1**

MAQKEKSKGESKEKFWLYAFRDHLANLRAFSNCIETADVSGNGDYQLLVADGSKKLKVFGGTALQRELPLFGVPSAIASFYMSTNDAFNKPVIAVATGPYIFMYRNNKPLYRYMIPAVPIDAQESDIWKKLADGVYTVEDAVAKLESLLDSGVQTSSRTLELLLLDTEEERTDFVTRMSAIPLIQMDVATCMTSIPLETLEAEGTSCLVVGTEACFLYVLGAATMEVSLKVVLPSPPVFLIVAGCFAVDYRIIIACRDGRVYSIKHGHLHSAVIQPDAQPCAVARFGNLIAVATTANTLTYYNLKGKKQQSLFLPCPITNLTTITDPITGEDRGLVVALSNGEIRVLVGTQLLHVSLVYGTVTAMKFCRYGRADGALILVLQNGSLVVELLHRNADLTSSKKVETGPPPEQDVPIPVPFLSSVFTAQTSRERKYGADMYQLFQYDLSQLRLTAAKAYLEMVGSGAVPTELGNVTEENEEVAESSLRMNTVVQGLGPVFKVKVQLQNIGAAPLHAVRVVFCLSDDDMYRMPQQVFTIPTLLPSVPLSCEALVELVEGEVKGNAILVVASEPKSTNPLASTLVDLPEAELIEGL

ATGGCGCAGAAGGAAAAAAGCAAAGGGGAGTCGAAGGAGAAGTTCTGGTTGTACGCCTTCCGCGACCACCTCGCCAACCTGCGCGCTTTTTCGAACTGTATCGAGACGGCCGACGTCAGCGGCAACGGCGACTACCAGCTGCTCGTGGCAGATGGCAGCAAAAAACTAAAGGTCTTCGGCGGCACCGCCCTGCAACGCGAGTTGCCTCTCTTTGGCGTGCCGTCGGCGATCGCCTCCTTTTACATGAGCACCAATGACGCCTTCAACAAGCCAGTAATCGCAGTGGCGACGGGGCCGTACATCTTCATGTACCGCAACAACAAACCTCTCTATCGCTACATGATTCCCGCCGTCCCGATTGACGCGCAGGAGTCGGATATTTGGAAAAAGCTCGCTGACGGCGTCTACACCGTCGAGGACGCCGTGGCGAAGTTGGAGTCGCTGCTCGACTCAGGTGTGCAGACCTCGTCGCGGACGCTGGAGTTGCTGCTGCTAGACACGGAGGAGGAGCGGACTGACTTTGTGACGCGTATGAGCGCCATCCCGCTCATTCAGATGGATGTGGCGACTTGCATGACGTCAATCCCGCTGGAGACACTGGAGGCAGAAGGCACGAGCTGCCTGGTCGTGGGTACCGAGGCGTGCTTTCTCTATGTCTTGGGGGCCGCGACGATGGAGGTGTCGCTGAAGGTGGTGCTGCCGAGCCCGCCGGTGTTTTTAATCGTGGCCGGCTGCTTTGCCGTGGATTACCGCATCATCATCGCATGTCGCGACGGCCGCGTTTACTCCATCAAGCACGGCCACCTGCACAGCGCTGTCATCCAGCCTGACGCGCAGCCTTGCGCCGTGGCTCGCTTCGGCAACTTGATCGCCGTTGCCACCACCGCCAACACGCTCACCTACTACAATCTGAAGGGCAAGAAGCAGCAAAGTCTGTTCCTGCCGTGCCCCATCACGAACTTGACCACCATCACTGACCCGATCACTGGAGAGGACAGGGGCCTCGTCGTCGCCCTCAGCAATGGCGAGATTCGCGTGCTGGTCGGCACGCAGCTGCTGCACGTGAGCCTCGTGTATGGTACGGTGACAGCCATGAAGTTCTGCCGCTACGGCCGCGCGGACGGGGCCCTCATTCTCGTCCTGCAGAACGGCTCGCTCGTCGTCGAGCTGCTGCACCGCAACGCCGACCTCACCTCCAGCAAAAAGGTGGAGACGGGCCCGCCACCGGAGCAGGACGTGCCGATCCCCGTGCCCTTTCTCAGCTCTGTCTTTACGGCGCAGACGTCGCGGGAGCGCAAGTACGGCGCGGACATGTACCAGCTTTTCCAGTACGACCTCTCGCAGCTGCGCTTGACAGCGGCAAAGGCGTATTTGGAGATGGTCGGTAGCGGCGCGGTGCCGACGGAGCTGGGCAACGTGACGGAGGAGAACGAGGAGGTGGCGGAGTCGTCCCTGCGCATGAATACTGTGGTGCAGGGGCTTGGGCCCGTCTTTAAGGTGAAGGTACAGCTGCAGAACATCGGTGCGGCGCCGCTGCATGCCGTCCGGGTGGTCTTCTGCCTCTCCGACGACGATATGTATCGCATGCCGCAGCAAGTTTTCACGATTCCGACGTTGCTGCCCTCCGTTCCGCTGTCATGTGAGGCGCTGGTGGAGTTGGTGGAAGGGGAGGTCAAGGGCAACGCAATTTTGGTCGTCGCCTCAGAGCCGAAGAGCACCAATCCGCTTGCAAGCACGTTGGTAGACCTTCCCGAGGCGGAGCTGATTGAGGGGCTGTAA

**LpTULP**

MNPSTPPRPPNQPPHPHGSAASVRFQRTHVVENTGSPTAATTTDAAVAPVLPCTPPTVPLRHPSSLQKQKKPEELVSNDSASEAEDNPNAVIPAGLTVDAVHRAFGSAADASAPPGAGAGVGVGGGGGGGGGAHVAGGGSPSHGIGSSGGRRPGGIIMPTRKEGNDSVSMYSMSQSVLPLDPRERIYYRPRRHLLQCYVERKKQQGHSIASLFPGGHKSFQFFLEHTNDFVLAAVPRNAKSRAVVEDTSDGIGGRFYAVSKGVGNIVFTVNQQQLDNDSRSFVGKLSRRASGLEMVMFSEGDKATRKEIVVVLLENFEDTSRSSFTVVLPAIDAESGYIRPVEGGEVRFLRDGRGGTSVAGGSRAGIDAAMAESSEDEVEATMSTMTKTAPKFAVVDASATPQVGGTVEAPAMAEGAATSSQKKWRTHSLLAKEYRRDPRSPHIIVLKNKVPQWDGVLRGYKLDFHGRATKASEKNFQLVAASDPEKVVMLFGKQSEDRFALDFRYPLCGLQAAAIATTIMTARKMIK

ATGAACCCTTCCACACCACCGCGGCCGCCCAATCAGCCCCCACACCCGCACGGTAGCGCCGCTAGTGTGCGCTTCCAGCGCACTCACGTTGTCGAGAACACCGGAAGCCCGACTGCGGCCACCACCACCGATGCTGCAGTGGCTCCCGTCCTTCCGTGTACGCCGCCCACCGTTCCTTTACGCCACCCGTCCTCCCTGCAGAAACAAAAAAAGCCGGAGGAACTCGTCAGCAACGACTCAGCCAGCGAGGCCGAGGATAACCCGAACGCCGTCATCCCGGCAGGGCTGACGGTGGACGCGGTGCACCGCGCCTTTGGTAGCGCGGCGGATGCCTCCGCGCCACCTGGCGCTGGCGCTGGCGTTGGCGTTGGCGGTGGCGGTGGCGGTGGCGGTGGCGCGCATGTGGCCGGCGGCGGGTCGCCGTCTCATGGGATTGGCAGTAGCGGCGGCCGTCGGCCTGGTGGGATCATCATGCCGACCCGGAAAGAGGGGAACGACTCGGTCTCCATGTACAGCATGTCGCAGTCCGTCCTGCCACTGGACCCGCGAGAGCGCATCTATTACCGCCCGCGCCGTCACCTGCTCCAGTGCTACGTTGAGCGCAAAAAACAGCAGGGCCACAGCATCGCCTCCCTTTTCCCAGGTGGTCACAAGTCCTTTCAGTTTTTCCTCGAGCACACCAACGACTTTGTGCTCGCTGCCGTCCCACGCAATGCCAAGTCGCGCGCCGTGGTTGAGGACACGTCGGACGGCATCGGTGGCCGTTTCTACGCTGTCTCTAAAGGTGTCGGCAACATCGTCTTCACAGTTAATCAGCAGCAGCTGGACAATGACTCGCGCAGCTTTGTCGGCAAGTTGTCCCGGCGTGCGAGCGGGCTGGAGATGGTGATGTTCAGTGAAGGTGACAAGGCAACACGGAAGGAGATCGTGGTCGTGCTGCTCGAGAACTTCGAGGACACGAGCCGCTCGTCATTTACGGTGGTGCTGCCCGCCATCGACGCAGAGTCGGGCTACATCAGACCGGTGGAGGGTGGCGAGGTGAGGTTCCTGCGCGACGGCCGCGGCGGGACGTCGGTGGCCGGCGGCAGTCGCGCCGGCATCGACGCGGCGATGGCCGAGTCGTCCGAGGACGAGGTAGAAGCAACGATGTCGACGATGACGAAGACGGCACCGAAGTTTGCCGTTGTCGACGCGTCCGCCACGCCGCAGGTCGGTGGGACCGTCGAGGCCCCCGCAATGGCGGAAGGGGCCGCGACGAGCTCACAGAAAAAGTGGCGCACGCACAGCTTGCTGGCGAAGGAGTACCGCCGCGACCCGCGCAGCCCACACATCATCGTCTTGAAGAACAAGGTGCCTCAGTGGGATGGCGTGTTGCGGGGTTACAAGTTAGATTTCCACGGTCGTGCCACGAAGGCGAGCGAGAAGAACTTCCAGCTGGTCGCTGCGTCAGATCCTGAGAAGGTCGTGATGTTGTTTGGTAAGCAGAGCGAGGACCGGTTCGCCTTGGACTTCCGCTACCCGCTTTGTGGTTTGCAGGCTGCCGCGATTGCAACGACCATCATGACTGCTCGTAAGATGATCAAGTAA
