# Supplementary material for: Protein subcellular relocalization and function of duplicated flagellar calcium binding protein genes in honey bee trypanosomatid parasite
Source: PLoS Genet. 2024 Mar 4;20(3):e1011195. doi: 10.1371/journal.pgen.1011195 (PMC10939215; doi:10.1371/journal.pgen.1011195)
Supplement: Attachment — Submitted filename: Response to reviewers.docx [file pgen.1011195.s010.docx]

Reviewer #1: Overview

This study seeks to characterises the location, localisation-determinants and function of two tandemly-duplicated genes in the trypanosomatid parasite of bees, Lotmaria passim, with the aim of understanding more about the processes driving/maintaining gene duplication and diversification. These proteins are highly similar, with the main differences occurring towards the N terminus, close to the myristate and palmitate addition signals. The authors express tagged proteins, and protein chimeras to demonstrate that the paralogs have different localisation distributions and that this is primarily conferred by the N terminal sequences. By expressing the N terminal peptide of the flagellar-localised paralog fused to an enzyme that performs promiscuous biotinylation followed by streptavidin purification of biotinylated proteins and western blot analysis, the authors present evidence that flagellar localisation of LpFCaBP1 is done via the N-terminal 16 residues interacting with interacting with BBS1. The authors perform KO experiments to show that although an effect is observed in vitro, KO parasites can still infect bees, suggesting a non-essential role in vivo.

Although the claim that the differential distributions of the two proteins is (predominantly) conferred by the N terminal sequences is solid, differential localisation of paralogs is not new and several of the descriptions and conclusions in the text do not tie with the data as shown in the Figures. Most importantly, however, the authors give little functional insights into these proteins, and the functional significance, if any, of their different localisation distributions is not investigated.

Main issues:

Line 126: “The N-terminal 16 amino acids of LpFCaBP1 and LpFCaBP2 were sufficient to direct GFP to the flagellum and cell body, respectively (Fig. 2)”.

Although this statement is justified by the experiment fusing the LmFCaBP1/2(1-16) to GFP, the other data appears to be more complex than the authors suggest in the text. For example, while the LpFCaBP1N28/FCaBP2-GFP does indeed appear to reproduce the LpFCaBP1-GFP full length protein, the LpFCaBP1N16/FcaBP2 does not, as this latter chimera appears to be missing from the cell-body membrane altogether. Would this not suggest that there is an additional signal in LpFCaBP1(N16-28) that facilitates some distribution to the cell-body membrane?

Additionally, both of the other chimeras (LpFCaBP2N28/FCaBP1-GFP and LpFCaBP2N16/FCaBP1-GFP) are missing from the flagellar membrane (at least in the example given), in contrast to FCaBP2-GFP. Would this not suggest that there are sequences in FCaBP2(16-204) that confer at least some flagellar membrane localisation?

Authors: We determined the ratio of mean fluorescence in the flagellum to that in the cell body using Image-J. There are no statistical differences (by the Steel test) between the ratios for LpFCaBP1-GFP (2.4 in average, Fig. 1), LpFCaBP1N28/FCaBP2-GFP (2.3 in average, Fig. 2), LpFCaBP1N16/FCaBP2-GFP (2.3 in average, Fig. 2), and LpFCaBP1N16-GFP (2.1 in average, Fig. 2). These results suggest that the N-terminal 16 amino acids of LpFCaBP1 are sufficient to direct GFP to the flagellum. The ratio for LpFCaBP2-GFP (0.92 in average, Fig. 1) is significantly higher than those of LpFCaBP2N28/FCaBP1-GFP (0.66 in average, P < 0.003, Fig. 2), LpFCaBP2N16/FCaBP1-GFP (0.78 in average, P < 0.04, Fig. 2), and LpFCaBP2N16-GFP (0.49 in average, P < 0.001, Fig. 2). These results suggest that EF-hand calcium binding domains of LpFCaBP2 (17-204) may contribute to the flagellar localization. These have been explained in the revised manuscript (Line 122-133 in yellow).

Line 152 – 170

the authors express the N terminal region of a series of calmodulin homologs from different trypanosomatid species and note different localisation distributions. In particular, they note that expressing the N24 residues of Tb/TcFCaBP localises to the cell body membrane, and not the flagellar membrane as has been shown for the full length protein in T. brucei/cruzi. From this they conclude that a the default localisation is the cell body membrane, and that a trans-acting factor causes localisation to the flagellar membrane. Beyond showing that the N terminal peptide clearly causes differences in localisation distribution (already known from Figure 2) I am unconvinced by this line of experiments and the conclusions that were drawn because of the biological irrelevance of drawing conclusions when expressing orthologs from diverse species and lack of controls (e.g. expression in native species, expression of full length protein). A more useful line of enquiry would have been to use these “natural mutation experiment” as a basis to perform directed mutagenesis to obtain mechanistic insight into the different sorting signals.

Authors: The results of Figs. 1 and 2 indicate that the N-terminal 16 amino acids of the LpFCaBP1 and LpFCaBP2 could direct GFP to flagellum and cell body membrane, respectively. The EF-hand calcium binding domains of LpFCaBP2 also contribute to the protein localization in the flagellum of *L. passim*. In Maric et al., paper (J. Biol. Chem., 286, 33109-33117, 2011), the authors showed the N-terminal 24 but not 12 amino acids of the TcFCaBP were sufficient to direct GFP to the flagellum of *T. cruzi*. These results prompted us to test how **the N-terminal flagellar and cell body membrane sorting sequences of FCaBPs** have conserved and changed during evolution of various trypanosomatid parasites. Because flagellar proteins depend on the specific machinery for the flagellar localization (IFT), we could learn about evolution of IFT (*trans*-acting factors) as well. Meanwhile, the EF-hand calcium binding domains of FCaBPs have been well conserved among the trypanosomatid parasites we tested (Supplementary data 1). We agree testing the functions of these sorting sequences in the native species is important; however, it is not possible to test the trypanosomatid parasites of interest, particularly human pathogens, Trypanosoma and Leishmania. We are not allowed to use them for research at our university.

To respond to above comments as well as those by the Reviewer #2, we performed targeted mutagenesis on LpFCaBP1 and LpFCaBP2. Specifically, lysine at position 11 (K11) in LpFCaBP1 was substituted with isoleucine, and vice versa, generating LpFCaBP1N16(K11I)-GFP and LpFCaBP2N16(I11K)-GFP. Additionally, serines at positions 9 (S9) and 10 (S10) in LpFCaBP1 were substituted with threonine and glutamine, respectively, and vice versa, resulting in LpFCaBP1N16-TQ-GFP and LpFCaBP2N16-SS-GFP. Comparative analyses with wild-type LpFCaBP1N16-GFP (Fig. 2) revealed a substantial increase in cell body fluorescence and a corresponding decrease in relative flagellar fluorescence with LpFCaBP1N16-TQ-GFP (average 1.2, *P < 0.001*), while no significant change was observed with LpFCaBP1N16(K11I)-GFP (Fig. 4B). In comparison to wild-type LpFCaBP2N16-GFP (Fig. 2), LpFCaBP2N16(I11K)-GFP exhibited an increase in relative flagellar fluorescence (average 0.79, *P < 0.001*), while no significant difference was noted with LpFCaBP2N16-SS-GFP (Fig. 4B). These results indicate that K11 of LpFCaBP1 contributes to direct GFP to the flagellum, although it is not essential. Furthermore, S9 and S10, which are conserved among FCaBP1s across four trypanosomatid species (Supplementary data 1), are crucial for the efficient localization of GFP to the flagellum in *L. passim*. Remarkably, the fluorescence pattern of LpFCaBP1N16-TQ-GFP and LpFCaBP2N16-SS-GFP is uniform within the cell body, akin to GFP (Fig. 1), in contrast to the membrane localization observed with LpFCaBP2N16-GFP (Fig. 2) and other GFP fusion proteins (Fig. 4B and C). Thus, it is inferred that S9, S10, T9, and Q10 are requisite for the efficient acylation at glycine (G2) and cysteine (C3) in the N-termini of FCaBP1 and FCaBP2. These lipid modifications appear to play a crucial role in determining the localization of FCaBP in either the flagellum or cell body membrane. These have been added in the revised manuscript (Line 176-215, Line 317-322, Line 377-381, and Line 715-716 in yellow).

Figure 5 and line 171-186.

The authors show that BBS1 appears to be somewhat biotinylated by LpFCaBP1N16-UltraID. However, the authors do not show that the an interaction of LpFCaBP1N16 (or, indeed, LpFCaBP1N16) with the BBSome is necessary for its flagellar localisation, for example with BBS KO cells. We should also note that this experiment was only done with the N terminal peptide, which is less biologically realistic than the full length protein

Although using Flag-LpTULP adds an important control, the BBS1 band is not very convincing and this data would be strengthened using label-free using mass spectrometry to confirm BBS1 presence and relative abundance.

Note that biotinylation does not necessarily indicate “interaction” but could simply indicate proximity.

I would advise the authors to investigate any interaction of the BBSome with LpFCaBP2, because the BBSome has been implicated in removal of proteins from the flagellum

Authors: We agree that it is important to test the localization of LpFCaBP1N16-GFP in *L. passim* mutant for BBSome. However, we found deleting *LpBBS1* gene is lethal in *L. passim* so that we were not able to directly test whether BBSome is required for the flagellar localization of LpFCaBP1N16-GFP. A method for conditional gene knock-out has to be developed with this parasite to address the above question. We also tried to establish *L. passim* stably expressing an ultraID fusion protein with either full length LpFCaBP1 or LpFCaBP2; however, it has never been successful despite of the multiple attempts. Expression of the above ultraID fusion proteins appear to be toxic in *L. passim*, and thus we depended on using the ultraID fusion protein with the N-terminal sorting sequence of LpFCaBP1 or LpFCaBP2.

Because it is very difficult for us to run label-free protein quantification based on mass spectrometry, we repeated the experiments with larger scale (using 4 × 10^8^ parasites). Furthermore, we tested whether the ultraID fusion protein with N-terminal cell body membrane sorting sequence of LpFCaBP2 (LpFCaBP2N16-UltraID) is in close proximity to either Flag-LpBBS1 or Flag-LpTULP. To our surprise, LpFCaBP2N16-UltraID concentrated at the anterior end of cell body (Fig. 5A) in contrast to the uniform cell body membrane localization of LpFCaBP2N16-GFP (Fig. 2). Thus, ultraID tends to anchor at the anterior side of cell body and this property may also inhibit the transport of LpFCaBP1N16-UltraID to the distal part of flagellum in *L. passim* (Fig. 5A). We could clearly detect biotinylated Flag-LpBBS1 but not Flag-LpTULP in the parasites expressing LpFCaBP1N16- but not LpFCaBP2N16-UltraID (Fig. 5B), suggesting that LpFCaBP1N16 is in close proximity to LpBBS1. These have been explained in the revised manuscript (Line 26-29, Line 216-234, Line 327-329, Line 406-410, and Line 420-422 in yellow).

Figure 7 and line 187-214:

The authors investigate the functional characterises of KO parasites. Here I think the authors have missed a trick. Given the emphasis on understanding paralogs with different localisations, performing a addback with the different paralogs individually would seem to be a good way of getting at their potential different function.

Additionally, further analysis of the KO parasites seem warranted, for example performing TEM on flagellar cross sections to detect defects, and a microscopical analysis of the KO parasites from in vivo infections, as a PCR-based assay seems to be rather coarse and may have missed a phenotype.

Authors: We also established *LpFCaBP1/2 -/-* parasites, wherein either *LpFCaBP1* or *LpFCaBP2* was introduced on a plasmid DNA vector containing a bleomycin resistance gene. Comparative phenotypic analyses were performed with wild-type and the parent mutant parasites at 29 ℃. *LpFCaBP1/2 -/-* parasites exhibited reduced growth rates during the logarithmic phase, reaching stationary phase at a density comparable to wild-type parasites. The growth defect in *LpFCaBP1/2 -/-* parasites was rescued by the introduction of either LpFCaBP1 or LpFCaBP2 (Fig. 7A and B). In the early stationary phase (at 60 h after culture initiation), wild-type parasites displayed active movement with extended flagella. Measurement of flagella and cell body lengths, as well as individual parasite movements, revealed that *LpFCaBP1/2 -/-* parasites exhibited shorter flagella and cell bodies with reduced motility compared to wild-type parasites (Fig. 7C-F and Supplementary videos 1 and 2). These findings indicate the essential role of LpFCaBP1 and LpFCaBP2 in flagellar morphogenesis and motility of *L. passim*. While either LpFCaBP1 or LpFCaBP2 rescued the observed phenotypes, they did not fully complement to wild-type levels, except for LpFCaBP1, which fully restored the short flagella of *LpFCaBP1/2 -/-* parasites (Fig. 7C-F and Supplementary videos 1-4). In the late stationary phase (at 96 h after culture initiation), wild-type parasites formed rosettes, characterized by clusters of cells with flagella directed towards the center. However, *LpFCaBP1/2 -/-* parasites remained as individual cells without clustering at the culture plate bottom (Fig. 7G). Both LpFCaBP1 and LpFCaBP2 restored rosette formation in *LpFCaBP1/2 -/-* parasites, although the sizes of rosettes were generally smaller than those observed with wild-type parasites (Fig. 7G). These results suggest that the primary function of LpFCaBP1 and LpFCaBP2 is the same as a calcium-binding protein; nevertheless, *L. passim* requires both LpFCaBP1 concentrated in the flagellum and LpFCaBP2 uniformly present in the entire parasite. These have been added in the revised manuscript (Line 252-276, Line 343-346, Line 492-503, and Line 748-771 in yellow).

We agree characterizing the flagellar defects of *LpFCaBP1/2 -/-* parasites in detail is valuable. However, TEM is not available at our university so that it is difficult to carry out the experiments. We observed the honey bee hindgut infected by either wild-type or *LpFCaBP1/2 -/-* parasites for 14 days by a light microscope. Both parasites formed the dense layer of spheroid/haptomonad in the hindgut and they were morphologically indistinguishable as shown in a figure below.

Minor points:

Line 115: “These findings indicate that the two duplicated FCaBP genes encode proteins with distinct subcellular localizations”.

The localisations are actually the same (flagellar membrane and cell-body membrane), but the key factor is their relative enrichment on each plasma-membrane sub-domain. Suggest to re-phrase as “differentially enriched, but overlapping, distributions”, or something that conveys the same idea

Authors: The sentence has been changed to “These findings indicate that the two duplicated *FCaBP* genes encode proteins enriched in the different part of parasite body with common localization in the flagellum” in the revised manuscript (Line 105-115 in yellow).

The miscopy figures all need scale bars. In several instances the cells appear to be different magnifications – would recommend standardising the magnification within a Figure.

Authors: We have added scale bars in the figures of revised manuscript. There are variations in sizes of flagella and cell bodies with individual parasites.

Unclear in some cases if it DOES localise a cell-body/flagellar membrane subdomain, but is difficult to discern because of the high levels of expression of the other subdomain (e.g. Figure 1 bottom two rows, but also perhaps in other places where there is strong expression to a particular subdomain). Can deal with this in a multiplicity of ways, but some quantification would be helpful

Authors: As explained above, we have determined the ratio of mean fluorescence in the flagellum to that of the cell body using Image-J. The mean value ± SD with the number of parasites analyzed has been added to Figures 1, 2, and 4 (Line 392-395, Line 684-686, Line 692-694, and Line 717-719 of the revised manuscript in yellow).

Figure 1:

FCaBP2-GFP needs a better example exhibiting a clear edge effect to show it is on the flagellar membrane

Provide clearer annotation for the unfamiliar reader to point out the flagellar membrane and cell body membrane

Authors: The panels for LpFCaBP2-GFP have been replaced and annotations for flagellum and cell body have been added in Figure 1 of the revised manuscript.

Figure 6

Can the authors clarify whether the screening primers are specific to each paralog?

Authors: The sequences of two screening primers, LpFCaBP-152R and LpFCaBP-538F, are common between *LpFCaBP1* and *2* genes. Since the two genes are separated by 1063 bp intergenic region (purple in Fig. 6A), the specific amplicons for 5’ and 3’ WT alleles (800 and 729 bp) were obtained by setting the extension time for PCR to 40 seconds. Indeed, there are only single bands for 5’ and 3’ WT alleles in Fig. 6B. Two primers used for the RT-PCR (Fig. 6C), LpFCaBP1-36R and LpFCaBP2-36R, are specific to each paralog. This was confirmed by PCR using the plasmid DNA carrying either *LpFCaBP1* or *LpFCaBP2* as a template and the upstream forward primer (based on the plasmid vector DNA sequence) as well as one of the above two reverse primers.

Figure 7A and B:

Clarify on whether the growth curve data and statistics are based on technical or biological replicates. If technical replicates (i.e. the same experiment done in triplicate) then the very mild phenotype observed could be simply due to differences in the starting “health” of the cultures, and not reproducible.

Clarify how the length of the flagellum was calculated (e.g. measured from phase images?)

Authors: The experiments were conducted by biological replicates. This has been described in the legend of Figure 7 in the revised manuscript (Line 752). The length of flagellum was measured with the phase images using image-J. This has been explained in the Materials and Methods section of revised manuscript (Line 505-507).

Reviewer #2: In the trypanosomatid parasites gene duplications are common and likely represent the evolution of proteins with novel functions. In this manuscript the authors investigate the duplication of a calcium binding protein in Lotmaria passim. They demonstrate that one protein localises to the flagellum and the other to the cell body and that one or the other are required for flagellum assembly and function. Overall, the cell biology data presented is of a high quality and generally supports the conclusions the authors make; however, the evolutionary analysis focussed on a restricted range of organisms, with limited discussion.

Abstract ln 30/31 – the authors do not present any data to show that the Tbrucei and Ldonovani FCaBPs do not interact with the BBSome. These leader sequences do not direct GFP to the Lpassim flagellum but that might not be due to the lack of BBSome interaction. Therefore, it is a stretch to conclude that this is due specific co-evolution between the Lotmaria BBSome and FCaBP. To really conclude this, they would need to demonstrate interaction of paired FCaBPs with BBSomes i.e. does Tbrucei FCaBP interact with Tbrucei BBSome in Tbrucei.

Authors: We agree testing the functions of these N-terminal sorting sequences and their interactions with BBSome complexes in the native species is important; however, it is not possible to test the trypanosomatid parasites of interest, particularly human pathogen, *Trypanosoma brucei*. We are not allowed to use it for research at our university. We have therefore deleted the sentence “These results suggest that the N-terminal flagellar sorting sequence of FCaBP1 has co-evolved with the BBSome complex in each trypanosomatid species.” in the revised manuscript.

This point leads onto my concern with the genome/sequence analysis in this paper, which could be improved. The authors look at the conservation/synteny etc. in a range of organisms but these are all relatively closely related – are FCaBP found in more distantly related organisms such as Paratrypanosoma or Bodo saltans? If so, does that help refine the relationship between the different species? Have the authors examined the phylogenetic relationships between these genes? Do they match the speciation of these organisms? Can this help to inform the analysis?

Authors: We searched *Bodo saltans* and *Paratrypanosoma confusum* genome sequences and found that *B. saltans* has two FCaBPs (BsFCaBP-A and B) and *P. confusum* contains one FCaBP (PcFCaBP). We used MUSCLE v5 program to align the amino acid sequences of 21 FCaBPs in total. The aligned sequence was then trimmed using trimA1 tool. Following the trimming, we built a phylogenetic tree via maximum likelihood method using IQ-TREE 2 (a tree below). The best-fit amino acid substitution models were selected by applying the ModelFinder program, and Q.pfam+G4 was chosen according to Bayesian information criterion. The bootstrap value was estimated using ultrafast bootstrap approximation with 10,000 replications in IQ-TREE 2.

The topology of tree is not consistent with those of trees constructed before (Flegontov et al., Curr. Biol. 23, 1787–1793, 2013; Simpson et al., Trend. Parasitol. 22, 168-174, 2006; Schwarz et al., J. Eukaryot. Microbiol. 62, 567–583, 2015). Bootstrap values are low at many branches due to the high conservation of FCaBP amino acid sequences. Thus, the phylogenetic tree based on FCaBPs does not appear to be helpful to refine the relationship between the different trypanosomatid species.

The authors rely on the reference genomes for these organisms as presented but are they sure that the loss of FCaBP2 in Lseymouri is real and not an issue with genome assembly/annotation – is there a pseudogene downstream of FCaBP1 in this organism? In Lmexicana etc FCaBP2 is described as a pseudogene but the genome describes this as a normal protein-coding gene just without the N-terminus found in other species and the transcriptomic evidence suggests it is expressed. This protein may be perfectly functional and in fact might have a different localisation and represent a specific evolution event in these species. Where does this protein localise? In Lmajor both genes appear to pseudogenes but is this backed up by transcriptomic datasets? I would urge caution of taking what is presented in genomes at face value, especially if it looks different to what is seen in other organisms – it maybe true but might be an issue with the automated assembly of the genome.

Authors: At downstream of *LsFCaBP1*, there is 173 bp genomic sequence capable of encoding a protein with low similarity to C-terminal 56 amino acid of FCaBP (e-value: 0.013) if translated. This may represent the remnant of *LsFCaBP2* pseudogene. We primarily characterized *L. seymouri* assembled genome sequence derived from the strain ATCC 30220 Lsey_0068 (Accession number: LJSK01000068); however, FCaBP genomic sequence is the same in another independently assembled genome sequence derived from the different strain, BHU1095 (Accession number: ANAF02000031). Thus, the loss of *LsFCaBP2* is unlikely to be the error of genome assembly. We wanted to directly analyze *L. seymouri* genomic DNA with *FCaBP*; however, it was not possible to import the strain ATCC 30220 Lsey_0068 from ATCC to China. These have been described in the revised manuscript (Line 159-166).

It is impossible to distinguish the normal (Query below) and short versions (Sbjct below with a stop codon in 5’ end) of *FCaBP* mRNAs in transcriptome data since their sequences are almost identical, for example, in *L. donovani* as shown below.

Query 181 ATGGAGGAGGTCTACCAGGGCTGTGTGAGCATCCTCCAGCTCGAGGAGTTTACAACGCGC 240

||||||||||||||||||||||||||||||||||||||||||||||||||||||||||||

Sbjct 1	ATGGAGGAGGTCTACCAGGGCTGTGTGAGCATCCTCCAGCTCGAGGAGTTTACAACGCGC 60

Query 241 CTGCGCAGCATCGTGAAACGTGCCTTCACTAAGGCAAAGAGCATGGGCAACACCGCCGGC 300

||||||||||||||||||||||||||||||||||||||||||||||||||||||||||||

Sbjct 61	CTGCGCAGCATCGTGAAACGTGCCTTCACTAAGGCAAAGAGCATGGGCAACACCGCCGGC 120

Query 301 GTCGGCCAATGCGACGACGAGTTTGTCGAATTTCTTGAGTTTCGGCTGATGCTGTGCTAC 360

||||||||||||||||||||||||||||||||||||||||||||||||||||||||||||

Sbjct 121 GTCGGCCAATGCGACGACGAGTTTGTCGAATTTCTTGAGTTTCGGCTGATGCTGTGCTAC 180

Query 361 ATATACGACTACCTCGAGCTCACCGTCATGTTTGACGAGATCGACACCTCCGGCAACATG 420

||||||||||||||||||||||||||||||||||||||||||||||||||||||||||||

Sbjct 181 ATATACGACTACCTCGAGCTCACCGTCATGTTTGACGAGATCGACACCTCCGGCAACATG 240

Query 421 CTGGTGGACGCGAGGGAGTTCAAGGCCGCCGTGCCGAAGATGGGTGAGTGGGGCCTTGTC 480

||||||||||||||||||||||||||||||||||||||||||||||||||||||||||||

Sbjct 241 CTGGTGGACGCGAGGGAGTTCAAGGCCGCCGTGCCGAAGATGGGTGAGTGGGGCCTTGTC 300

Query 481 ATTGAGGATCCGGACACAATCTTCAAGGAAATCGACGACAACGGCTCCGGCCAGGTGTCC 540

||||||||||||||||||||||||||||||||||||||||||||||||||||||||| ||

Sbjct 301 ATTGAGGATCCGGACACAATCTTCAAGGAAATCGACGACAACGGCTCCGGCCAGGTGCCC 360

Query 541 TTTGACGAGCTCGCTGCGTGGGCCA 565

|||||||||||||||||||||||||

Sbjct 361 TTTGACGAGCTCGCTGCGTGGGCCA 385

If the short version of *FCaBP* is translated, it would be localized in cytosol but not membrane due to the lack of N-terminal glycine and cysteine residues for myristoylation and palmitoylation. Although the protein should be able to bind calcium, its interactions with other membrane associated proteins as a signaling factor would be lost. This has been explained in the revised manuscript (Line 146-151).

We searched for *LmFCaBP1* and *2* mRNAs (with multiple internal stop codons) in several RNA-seq data of *L. major* and found that there are the corresponding reads. However, they would represent a polycistronic mRNA precursor containing *LmFCaBP* sequences but not the mature mRNA with splice leader and 3’ poly A tail. Indeed, we did not find *LmFCaBP* reads in RNA-seq data with low sequencing depth, for example, SRX368109 (157.6M bases). Three independently assembled genome sequences are available for *L. major* (strains Friedlin, SD75.1, and LV39c5) and all of annotated *LmFCaBP* genes contain the multiple internal stop codons, suggesting that this is not the result of assembly error. These have been described in the revised manuscript (Line 153-156).

Does the alignment of these leader sequences suggest what is important for flagellum retention/exclusion – the dipeptide SS – TQ could be potentially important? Is this a charge effect there are lots of lysines, which are supposed to be important for flagellum localisation in Tbrucei/Tcruzi but those that do not localise to the Lpassim flagellum have a number of asparagine/glutamine residues in the leader sequence – is there any pattern in the overall charge of these sequences and their localisation?

Authors: The N-terminal sorting sequences are rich with lysine for both FCaBP1 and 2. Nevertheless, lysine 11 (K11) appears to be conserved between FCaBP1s of four species, *C. fasciculata, L. pyrrhocoris L. seymouri,* and *L. passim* (Supplementary data 1)*.* Thus, we performed targeted mutagenesis on LpFCaBP1 and LpFCaBP2. Specifically, lysine at position 11 (K11) in LpFCaBP1 was substituted with isoleucine, and vice versa, generating LpFCaBP1N16(K11I)-GFP and LpFCaBP2N16(I11K)-GFP. Additionally, serines at positions 9 (S9) and 10 (S10) in LpFCaBP1 were substituted with threonine and glutamine, respectively, and vice versa, resulting in LpFCaBP1N16-TQ-GFP and LpFCaBP2N16-SS-GFP. Comparative analyses with wild-type LpFCaBP1N16-GFP (Fig. 2) revealed a substantial increase in cell body fluorescence and a corresponding decrease in relative flagellar fluorescence with LpFCaBP1N16-TQ-GFP (average 1.2, *P < 0.001*), while no significant change was observed with LpFCaBP1N16(K11I)-GFP (Fig. 4B). In comparison to wild-type LpFCaBP2N16-GFP (Fig. 2), LpFCaBP2N16(I11K)-GFP exhibited an increase in relative flagellar fluorescence (average 0.79, *P < 0.001*), while no significant difference was noted with LpFCaBP2N16-SS-GFP (Fig. 4B). These results indicate that K11 of LpFCaBP1 contributes to direct GFP to the flagellum, although it is not essential. Furthermore, S9 and S10, which are conserved among FCaBP1s across four trypanosomatid species (Supplementary data 1), are crucial for the efficient localization of GFP to the flagellum in *L. passim*. Remarkably, the fluorescence pattern of LpFCaBP1N16-TQ-GFP and LpFCaBP2N16-SS-GFP is uniform within the cell body, akin to GFP (Fig. 1), in contrast to the membrane localization observed with LpFCaBP2N16-GFP (Fig. 2) and other GFP fusion proteins (Fig. 4B and C). Thus, it is inferred that S9, S10, T9, and Q10 are requisite for the efficient acylation at glycine (G2) and cysteine (C3) in the N-termini of FCaBP1 and FCaBP2. These lipid modifications appear to play a crucial role in determining the localization of FCaBP in either the flagellum or cell body membrane.ig. 4). Thus, S9 as well as S10 and T9 as well as Q10 would be required for the efficient acylation at glycine and cysteine in the N-termini of FCaBP1 and FCaBP2, respectively. These lipid modifications appear to be important for the localization of FCaBP in either flagellum or cell body membrane. These have been added in the revised manuscript (Line 176-200, Line 317-322, and Line 377-381).

Minor comments

Need to be specific with descriptions of localisation – presumably the proteins localise to the flagellum membrane and cell body membrane, not the cell body.

Authors: With the images of *L. passim* expressing various GFP fusion proteins, it is difficult to confirm the flagellar membrane localization. However, this is possible for cell body membrane so that we have changed “cell body” to “cell body membrane” where appropriate throughout the revised manuscript.

The localisation of PFR5 shown here is not how a tagged PFR protein would normally look like. Is the PFR discontinuous in Lotmaria?

Authors: Localization of LpPFRP5-GFP fusion protein was not uniform in the flagellum of *L. passim* (Fig. 1). Because PFR has never been studied with *L. passim*, it is not possible to tell whether this is true or artefact of the ectopic expression of the fusion protein at this stage.

Can you include geneIDs in the figure legends so it is easier to find the genes they are examining.

Authors: The gene IDs (accession numbers) have been added in the legend of Figure 4 in the revised manuscript (Line 709-712). We annotated *C. fasciculata* and *L. passim* *FCaBPs* from the genome sequences. All FCaBP sequences analyzed in this manuscript are available in Supplementary data 1.

What do the colours represent in figure 3?

Authors: *FCaBP1* and *2* genes (red and blue arrows) are flanked by three novel genes (beige, light grey, and light green arrows), as well as dynein light chain (light blue arrow) and sucrose-phosphate synthase-like protein genes (dark grey arrow) (Line 702-704).

Ln169/170 – the authors suggest that flagellum localisation requires sorting into the flagellum but this could equally be about retrieval of FCaBP2 from the flagellum – additional nuances should be discussed about what might define the localisation of a protein.

Authors: The sentence “These results suggest that dually acylated FCaBP is targeted to the cell body membrane by default, and efficient flagellar localization requires trans-acting factor(s) to recognize the specific flagellar sorting sequence.” has been deleted in the revised manuscript.

There was a limited information about the blots in figure 5. What was the loading of each lane, how many cell equivalents? What is the relationship between the input and streptavidin material? Without this information it is hard to judge how strong/likely this interaction is. In the discussion, there is a caveat about the weak signal detected here. Additional evidence to show this was a reciprocal interaction with FCaBP1 detected when BBSome1 was tagged with ultraID would increase confidence in this conclusion.

Authors: The input represents the total amount of Flag-LpBBS1 or Flag-LpTULP1 protein in 2.5 × 10^7^ parasites and the streptoavidin represents the total amount of biotinylated Flag-LpBBS1 or Flag-LpTULP1 protein in 10^8^ parasites. Thus, there is 4-fold difference between the input and streptoavidin. The band intensity of biotinylated Flag-LpBBS1 significantly increased when the experiments were repeated with more parasites. These have been described in the Materials and Methods section as well as legend of Figure 5 in revised manuscript (Line 434-449 and Line 728-729).

We attempted to establish *L. passim* stably expressing LpBBS1-ultraID fusion protein; however, it has never been successful similar to LpBBS1-GFP fusion protein. BBS1 functions as BBSome complex together with other subunits and incorporating above fusion protein into the complex may interfere the functions.

The functional analysis used a cell line in which both FCaBP genes had been deleted. How many clones were examined? Can you add this information to the legends etc? By deleting both genes, it is not possible to determine the relative contribution of each gene to the phenotype. Have the authors deleted each gene individually? Could they add back FCaBP1 into the deletion line and see if this restores the phenotype? Without this, it is not possible to determine if these proteins actually have separate functions as would be suggested by the PSR hypothesis.

Authors: We first characterized growth in the culture medium, flagellar length, movement, and rosette formation with two independent clones of *LpFCaBP1/2 -/-* parasites (D1 and F6) and their phenotypes were the same. We thus used the clone D1 for further experiments. These have been described in the Materials and Methods section as well as legend of Figure 7 in the revised manuscript (Line 515-517 and Line 748-749).

Because it was difficult to individually delete either *LpFCaBP1* or *2* gene, we established *LpFCaBP1/2 -/-* parasites, wherein either *LpFCaBP1* or *LpFCaBP2* was introduced on a plasmid DNA vector containing a bleomycin resistance gene. Comparative phenotypic analyses were performed with wild-type and the parent mutant parasites at 29 ℃. *LpFCaBP1/2 -/-* parasites exhibited reduced growth rates during the logarithmic phase, reaching stationary phase at a density comparable to wild-type parasites. The growth defect in *LpFCaBP1/2 -/-* parasites was rescued by the introduction of either LpFCaBP1 or LpFCaBP2 (Fig. 7A and B). In the early stationary phase (at 60 h after culture initiation), wild-type parasites displayed active movement with extended flagella. Measurement of flagella and cell body lengths, as well as individual parasite movements, revealed that *LpFCaBP1/2 -/-* parasites exhibited shorter flagella and cell bodies with reduced motility compared to wild-type parasites (Fig. 7C-F and Supplementary videos 1 and 2). These findings indicate the essential role of LpFCaBP1 and LpFCaBP2 in flagellar morphogenesis and motility of *L. passim*. While either LpFCaBP1 or LpFCaBP2 rescued the observed phenotypes, they did not fully complement to wild-type levels, except for LpFCaBP1, which fully restored the short flagella of *LpFCaBP1/2 -/-* parasites (Fig. 7C-F and Supplementary videos 1-4). In the late stationary phase (at 96 h after culture initiation), wild-type parasites formed rosettes, characterized by clusters of cells with flagella directed towards the center. However, *LpFCaBP1/2 -/-* parasites remained as individual cells without clustering at the culture plate bottom (Fig. 7G). Both LpFCaBP1 and LpFCaBP2 restored rosette formation in *LpFCaBP1/2 -/-* parasites, although the sizes of rosettes were generally smaller than those observed with wild-type parasites (Fig. 7G). These results suggest that the primary function of LpFCaBP1 and LpFCaBP2 is the same as a calcium-binding protein; nevertheless, *L. passim* requires both LpFCaBP1 concentrated in the flagellum and LpFCaBP2 uniformly present in the entire parasite. These have been added in the revised manuscript (Line 252-276, Line 343-346, Line 492-503, and Line 748-771).

Ln261/262 – Lmajor and Lpassim have evolved separately for millions of years and have very different life cycles etc, so I think the dispensability of FCaBP in Lpassim is likely unrelated to the ability of Lmajor to survive without these genes.

Authors: The sentence has been changed to “We found that LpFCaBPs are not essential for the viability of *L. passim*.” in the revised manuscript (Line 333).
