# Supplementary material for: Protein subcellular relocalization and function of duplicated flagellar calcium binding protein genes in honey bee trypanosomatid parasite
Source: PLoS Genet. 2024 Mar 4;20(3):e1011195. doi: 10.1371/journal.pgen.1011195 (PMC10939215; doi:10.1371/journal.pgen.1011195)
Supplement: Attachment — Submitted filename: Response to Reviewer 2nd.docx [file pgen.1011195.s011.docx]

Reviewer #1: The authors have done a substantial amount of work and the manuscript is greatly improved.

Revisions:

Line 343: “We also found that either FCaBP1 or FCaBP2 alone can not fully rescue the phenotypes of LpFCaBPs-deleted parasites (Fig. 7), suggesting that L. passim depends on both proteins with the flagellar and entire body localization” The authors should acknowledge (or rule out if they have data to indicate otherwise, such as a western blot) that the inability to fully rescue the KO phenotype could be because the individual FCaBP1 or FCaBP2 addbacks do not express as highly as the combined FCaBP1/ FCaBP2 in the wild type.

Authors: We recognize the importance of this point. However, due to the lack of a specific antibody for LpFCaBP1/2, we are unable to directly compare the levels of ectopic and endogenous LpFCaBP1/2 proteins using western blot analysis. According to our previous study (Liu Q, Lei J, Kadowaki T. Gene Disruption of Honey Bee Trypanosomatid Parasite, Trypanosomatid Parasite, Lotmaria passim, by CRISPR/Cas9 System. Front Cell Infect Microbiol. 2019; 9:126), plasmid vectors carrying either *LpFCaBP1* or *LpFCaBP2* are expected to be present as multiple copies of episomal DNA in the *LpFCaBPs*-deleted parasite. Therefore, we anticipate that the levels of ectopically expressed FCaBP1/2 proteins will be comparable to those of GFP-fusion proteins shown in Fig. 1. Nevertheless, we have revised the sentence to state: "We also found that neither FCaBP1 nor FCaBP2 alone can fully rescue the phenotypes of *LpFCaBPs*-deleted parasites (Fig. 7). Assuming that the amount of ectopic FCaBP1 or FCaBP2 protein is comparable to that of the endogenous protein, *L. passim* relies on both proteins for flagellar and entire body localizations.” in the manuscript's second revision (Line 349-353).

The microscopy still needs scale bars

Authors: We added scale bars in the revised manuscript; however, we have modified to make them more visible in the manuscript's second revision.

Reviewer #2: On the whole the authors have suitably addressed my concerns. They have provided a more thorough genomic analysis and have provided additional functional data by examining LpFCaBP1/2 add back parasites to define their relative roles. However, while the inclusion of the enrichment analysis has improved the manuscript and helps to interpret their findings they need to more clearly explain the results and provide more details about the methodology.

More detail about measurement of fluorescence needed – was there a background correction? Was all the cell body/flagellum measured or just a part of it? What microscope was used? Was a consistent exposure time used etc.?

Authors: We subtracted the mean background fluorescence from the mean fluorescence of GFP in both the flagellum and cell body of individual parasites to determine the ratio. The mean fluorescence of GFP was measured in the entire flagellum and cell body. Further methodological details can be found in the Materials and Methods section of the second revision of the manuscript (Line 397-404).

Figure 1 - If the ratio of LpFCaBP2 between flagellum and cell body is 0.92±0.28 then it is essentially evenly distributed between the flagellum and cell body and at the moment the manuscript suggests that it is enriched in the cell body.

Authors: Yes, that is correct and we thus modified in the revised manuscript. Nevertheless, we have further checked and changed it where appropriate in the manuscript's second revision. (Line 20, 38, 120, 178-179, 202, 300, 319). As shown in Figure 2, N-terminal 16 amino acids of LpFCaBP2 function as cell body sorting sequence.

Figure 2 - For the LpFCaBP1N16/FCaBP2-GFP fusion how was the measurement of the flagellum fluorescence intensity performed was this the entire flagellum or just the proximal domain? On a related note this localisation pattern could represent an enrichment within the flagellar pocket and pocket neck membranes which constitute another membrane domain in these parasites, rather than the proximal domain of the flagellum.

Authors: We measured the mean GFP fluorescence in the entire flagellum and cell body, as described earlier. The GFP fluorescence seems to be weak in the distal part of the flagellum of parasite expressing LpFCaBP1N16/FCaBP2-GFP. However, this phenomenon was not observed in other parasites, where the fluorescence was generally uniform throughout the entire flagellum. Therefore, we have replaced these images with those of another parasite expressing LpFCaBP1N16/FCaBP2-GFP in the second revision of the manuscript.

Figure 2 - The authors need to describe the localisation pattern more clearly. The enrichment analysis shows no difference but the LpFCaBP1N16/FCaBP2-GFP shows an enrichment in the proximal flagellum region whereas the LpFCaBP1N28/FCaBP2-GFP is enriched along the entire flagellum as is the LpFCaBP1N16-GFP fusion and this is not pointed out. Moreover, as the LpFCaBP2N16-GFP gives a different enrichment ratio to LpFCaBP2-GFP the authors are correct in stating that the EF hands likely have an influence on protein localisation but this does not come across well in the text. At what point in the proteins does the EF hand domain start? This should be indicated somewhere in the manuscript.

Authors: As explained above, GFP fluorescence was generally uniform in the entire flagellum of parasite expressing LpFCaBP1N16/FCaBP2-GFP. The EF-hand calcium binding domains of LpFCaBP2 (amino acids 38-189) contribute to the flagellar localization. This has been described in the second revision of the manuscript (Line 129-134).

Line 146-9 – the authors should indicate that this protei n would likely be in the cytosol as they have no evidence to indicate its localisation.

Authors: The sentence has been revised to read: "If this gene is translated, the truncated protein lacking the N-terminal sorting sequence is synthesized and is likely to be present in the cytosol rather than the membrane, as it lacks the N-terminal glycine and cysteine residues necessary for myristoylation and palmitoylation." This change appears in the second revision of the manuscript (Line 147-151).

Figure 4 - As the SS/TQ mutants appear to be cytosolic the key conclusion which the authors make is that this seems to affect fatty acid modification but because of that the enrichment ratio is not overly informative as it is unlikely that the differential localisation is occurring through the same mechanism.

Authors: Since we measured the mean GFP fluorescence across the entire flagellum/cell body, the enrichment ratio was unable to discern differences in GFP localization within the cell body (membrane vs. cytosol). The absence of cell body membrane association of the SS/TQ mutants (as indicated by GFP localization) is likely due to defective acylation at the Glycine (G2) and Cysteine (C3) residues. Previous studies on *T. cruzi* and *T. brucei* FCaBPs have shown that acylation is crucial for flagellar localization (Maric D, McGwire BS, Buchanan KT, Olson CL, Emmer BT, Epting CL, et al. Molecular determinants of ciliary membrane localization of Trypanosoma cruzi flagellar calcium-binding protein. J Biol Chem. 2011;286(38):33109-17; Emmer BT, Souther C, Toriello KM, Olson CL, Epting CL, Engman DM. Identification of a palmitoyl acyltransferase required for protein sorting to the flagellar membrane. J Cell Sci. 2009;122(Pt 6):867-74.). This is consistent with the lack of enrichment of LpFCaBP1N16-TQ-GFP in the flagellum. However, it's possible that SS/TQ mutants failed to associate with the cell body membrane through another mechanism, as we don’t have direct evidence indicating impaired acylation of the mutant proteins. Therefore, the main text has been revised to state: "Thus, it is likely that S9, S10, T9, and Q10 are required for efficient acylation at glycine (G2) and cysteine (C3) in the N-termini of FCaBP1 and FCaBP2. These lipid modifications appear to play a crucial role in determining FCaBP localization, either in the flagellum as previously reported (17, 19) or in the entire body membrane. However, LpFCaBP1N16-TQ-GFP and LpFCaBP2N16-SS-GFP may fail to associate with the cell body membrane due to a mechanism other than defective acylation." This change is reflected in the second revision of the manuscript (Line 198-204).

Line 190 – the conclusion here needs to be modified as the results show that K11 is not necessary for flagellum localisation of LpFCaBP1N16. It appears therefore to contribute to LpFCaBP2N16 flagellum localisation but is not necessary for LpFCaBP1N16 flagellum localisation.

Authors: The sentence has been changed to “These results indicate that K11 of LpFCaBP1 is not essential to direct LpFCaBP1N16-GFP to the flagellum but contributes to the flagellar localization of LpFCaBP2N16-GFP.” in the second revision of the manuscript (Line 190-192).

Figure 4C – the authors point out the proximal flagellum enrichment of the Tc/Ld FCaBP leader sequences and again this relates to my comment about the flagellar pocket membrane domain that could be added to the discussion. The authors comment on the inefficient localisation (lines 298-300) but they should acknowledge the present of another membrane domain here as well.

Authors: The following sentence “Meanwhile, GFP may also exist in the flagellar pocket and pocket neck membranes in these parasites.” has been added in the Discussion section of the second revision of the manuscript (Line 304-305).

Line 274 – can the authors clarify this sentence, it is unclear what they mean by function is the same as a calcium binding protein.

Authors: The sentence has been revised to read: "These results suggest that the molecular functions of LpFCaBP1 and LpFCaBP2 remain the same as calcium-binding proteins. However, in *L. passim*, both LpFCaBP1 concentrated in the flagellum and LpFCaBP2 present throughout the entire parasite are necessary." This change is reflected in the second revision of the manuscript (Line 277-280).

Finally, the language of the paper needs to be improved throughout and the manuscript should be read by a native speaker before publication.

Authors: Unfortunately, there's currently no colleague with native English proficiency in the Department. Consequently, we have made efforts to enhance the language using AI-based editing tools.

Have all data underlying the figures and results presented in the manuscript been provided?

Large-scale datasets should be made available via a public repository as described in the PLOS Genetics data availability policy, and numerical data that underlies graphs or summary statistics should be provided in spreadsheet form as supporting information.

Reviewer #1: Yes

Reviewer #2: No: The numerical data for the graphs in figure 7 and enrichment analysis was not provided.

Authors: These data have been added as Supplementary data 4 and 5 in the second revision of the manuscript (Line 794-797).
